# Temporal dynamics in a red alga dominated geothermal feature in Yellowstone National Park

**DOI:** 10.1093/ismeco/ycae151

**Published:** 2024-12-03

**Authors:** Timothy G Stephens, Julia Van Etten, Timothy McDermott, William Christian, Martha Chaverra, James Gurney, Yongsung Lee, Hocheol Kim, Chung Hyun Cho, Erik Chovancek, Philipp Westhoff, Antonia Otte, Trent R Northen, Benjamin P Bowen, Katherine B Louie, Kerrie Barry, Igor V Grigoriev, Thomas Mock, Shao-Lun Liu, Shin-ya Miyagishima, Masafumi Yoshinaga, Andreas P M Weber, Hwan Su Yoon, Debashish Bhattacharya

**Affiliations:** Department of Biochemistry and Microbiology, Rutgers, The State University of New Jersey, New Brunswick, New Jersey 08901, United States; Graduate Program in Ecology and Evolution, Rutgers, The State University of New Jersey, New Brunswick, NJ, 08901, United States; Department of Land Resources and Environmental Sciences, Montana State University, Bozeman, MT 59717, United States; Department of Chemistry and Biochemistry, Montana State University, Bozeman, MT 59717, United States; Department of Microbiology and Cell Biology, Montana State University, Bozeman, MT 59717, United States; Department of Land Resources and Environmental Sciences, Montana State University, Bozeman, MT 59717, United States; Department of Biological Sciences, Sungkyunkwan University, Suwon 16419, Korea; Department of Biological Sciences, Sungkyunkwan University, Suwon 16419, Korea; Department of Biological Sciences, Sungkyunkwan University, Suwon 16419, Korea; Gregor Mendel Institute of Molecular Plant Biology, Vienna 1030, Austria; Institute of Plant Biochemistry, Cluster of Excellence on Plant Science (CEPLAS), Heinrich Heine University, 40225 Düsseldorf, Germany; Institute of Plant Biochemistry, Cluster of Excellence on Plant Science (CEPLAS), Heinrich Heine University, 40225 Düsseldorf, Germany; School of Environmental Sciences, University of East Anglia (UEA), Norwich Research Park, Norwich NR4 7TJUnited Kingdom; U.S. Department of Energy Joint Genome Institute, Lawrence Berkeley National Laboratory, Berkeley, CA 94720, United States; Environmental Genomics and Systems Biology Division, Lawrence Berkeley National Laboratory, Berkeley, CA 94720, United States; U.S. Department of Energy Joint Genome Institute, Lawrence Berkeley National Laboratory, Berkeley, CA 94720, United States; Environmental Genomics and Systems Biology Division, Lawrence Berkeley National Laboratory, Berkeley, CA 94720, United States; U.S. Department of Energy Joint Genome Institute, Lawrence Berkeley National Laboratory, Berkeley, CA 94720, United States; Environmental Genomics and Systems Biology Division, Lawrence Berkeley National Laboratory, Berkeley, CA 94720, United States; U.S. Department of Energy Joint Genome Institute, Lawrence Berkeley National Laboratory, Berkeley, CA 94720, United States; U.S. Department of Energy Joint Genome Institute, Lawrence Berkeley National Laboratory, Berkeley, CA 94720, United States; Environmental Genomics and Systems Biology Division, Lawrence Berkeley National Laboratory, Berkeley, CA 94720, United States; Department of Plant and Microbial Biology, University of California Berkeley, Berkeley, CA 94720, United States; School of Environmental Sciences, University of East Anglia (UEA), Norwich Research Park, Norwich NR4 7TJUnited Kingdom; Department of Life Science & Center for Ecology and Environment, Tunghai University, Taichung 40704, Taiwan; Department of Gene Function and Phenomics, National Institute of Genetics, Shizuoka 411-8540, Japan; Department of Genetics, The Graduate University for Advanced Studies (SOKENDAI), Shizuoka 411-8540, Japan; Department of Molecular and Cellular Biology, Kennesaw State University, Kennesaw, GA 30144, United States; Institute of Plant Biochemistry, Cluster of Excellence on Plant Science (CEPLAS), Heinrich Heine University, 40225 Düsseldorf, Germany; Department of Biological Sciences, Sungkyunkwan University, Suwon 16419, Korea; Department of Biochemistry and Microbiology, Rutgers, The State University of New Jersey, New Brunswick, New Jersey 08901, United States

**Keywords:** Yellowstone National Park, hot springs, cyanidiophyceae, extremophiles, community interactions, multi-omics, microbiome

## Abstract

Alga-dominated geothermal spring communities in Yellowstone National Park (YNP), USA, have been the focus of many studies, however, relatively little is known about the composition and community interactions which underpin these ecosystems. Our goal was to determine, in three neighboring yet distinct environments in Lemonade Creek, YNP, how cells cope with abiotic stressors over the diurnal cycle. All three environments are colonized by two photosynthetic lineages, *Cyanidioschyzon* and *Galdieria*, both of which are extremophilic Cyanidiophyceae red algae. *Cyanidioschyzon*, a highly specialized obligate photoautotroph, dominated cell counts at all three Lemonade Creek environments. The cell cycle of *Cyanidioschyzon* in YNP matched that observed in synchronized cultures, suggesting that light availability plays a strong role in constraining growth of this alga in its natural habitat. Surprisingly, the mixotrophic and physiologically more flexible *Galdieria*, was a minor component of these algal populations. Arsenic detoxification at Lemonade Creek occurred *via* complementary gene expression by different eukaryotic and prokaryotic lineages, consistent with this function being shared by the microbial community, rather than individual lineages completing the entire pathway. These results demonstrate the highly structured nature of these extreme habitats, particularly regarding arsenic detoxification.

## Introduction

Prokaryotes are often the only cells capable of surviving habitats with extremes of pH, temperature, solar radiation, salt concentrations, and atmospheric pressure. Whereas this is generally the case, particularly under the most extreme conditions, there are many lineages of eukaryotes that thrive in extreme environments. One such lineage is the unicellular photosynthetic red algae, Cyanidiophyceae [1, 2] which includes well-studied genera such as *Galdieria* and *Cyanidioschyzon* [3]. These cells inhabit, and often dominate, volcanic geothermal springs and acid mining sites characterized by extremes of light levels, relatively high temperature (35°C–56°C), and low pH (0 to 5), with high salt and toxic heavy metal concentrations [4, 5]. Several studies have identified Cyanidiophyceae in a range of acidic geothermal features in Yellowstone National Park (YNP), including the site studied here, Lemonade Creek [4, 6–8]. Analysis of 18S rRNA and *rbcL* gene data demonstrates that two algal genera, *Galdieria* and *Cyanidioschyzon*, are present in Lemonade Creek [6, 7, 9]. Analyses of sites at YNP, including Norris Geyser Basin indicates significant levels of mercury (Hg) in the soils as well as in springs [10–12], where it readily enters the food chain [11, 12], presumably *via* the algae in outflow channels [11, 12]. Arsenic is also prominent, occurring primarily as the more toxic arsenite [6, 7, 9, 13]. *Cyanidioschyzon* mats are capable of oxidizing arsenite to arsenate [14], which is a resistance and detoxification mechanism (arsenate is significantly less toxic). Furthermore, *Cyanidioschyzon* cultured from Norris Geyser Basin can methylate arsenite [As(III)] as a resistance mechanism using the enzyme ArsM, a *S*-adenosylmethionine (SAM) methyltransferase [13], whose encoding gene is highly expressed in situ by biofilms in Lemonade Creek [8].

**Figure 1:**
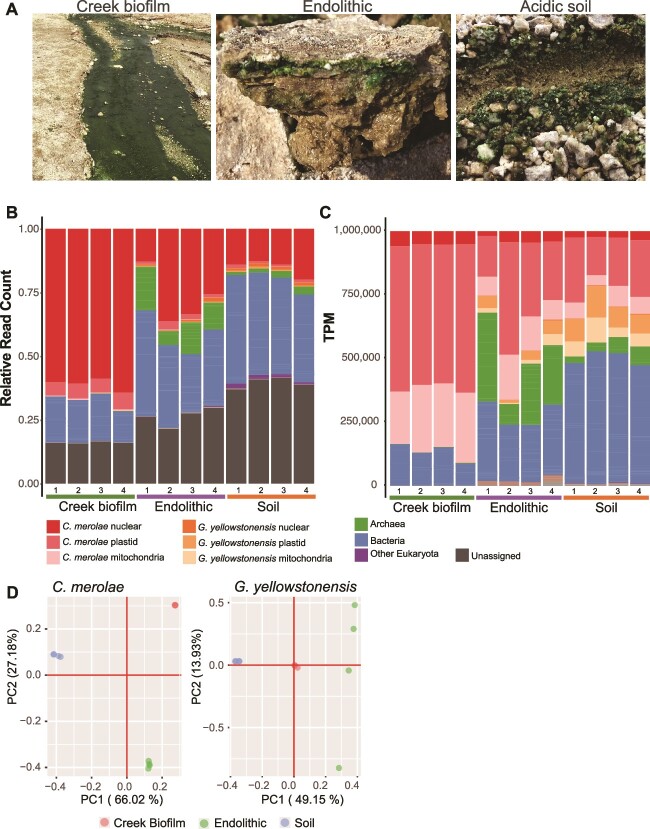
The studied YNP habitats and analysis of metagenome data. (A) The submerged creek biofilm, endolithic, and adjacent acidic soil habitats that were sampled in Lemonade Creek, YNP. (B) The relative number of reads (out of the total corrected reads from a given sample) that aligned to each type of MAG (i.e. bacterial, archaeal, eukaryotic, and unassigned) and the *C. merolae* 10D and *G. yellowstonensis* 5587.1 reference nuclear and organelle genomes in the combined non-redundant dataset from the three YNP habitats. (C) The relative abundance, in transcripts per million (TPM), of each MAG. (D) Principal component analysis (PCA) of *C. merolae* and *G. yellowstonensis* single-nucleotide polymorphism (SNP) data from the three studied habitats at YNP. The first two principal components are shown for each species. These data demonstrate that the algal populations have a high degree of distinctness in PC1. For *G. yellowstonensis,* the Endolithic populations are dispersed largely due to lack of SNP data from the Creek biofilm population (PC1, 0; PC2, 0).

Cyanidiophyceae, which are descended from mesophilic red algal ancestors, have adapted to life in geothermal springs due to horizontal gene transfer (HGT), which has led to ca. 1% of their nuclear gene inventory being comprised of HGT-derived prokaryotic genes [1]. These foreign sequences confer polyextremophily, including metal and xenobiotic resistance and detoxification (including mercury and arsenic, which are ubiquitous in these environments), cellular oxidant reduction, carbon metabolism, amino acid metabolism, osmotic resistance, and salt tolerance [1, 15–17]. Here, we used environmental multi-omics to investigate some of the biotic interactions that underpin the geothermal habitats at Lemonade Creek. Our goal was to decipher the roles of Cyanidiophyceae and prokaryotes in sustaining these communities in three distinct habitats: submerged lush biofilms, immediately adjacent endolithic sites, and acidic soils bordering the creek (Fig. 1A). Our sampling strategy also assessed community transcriptional and metabolic responses to solar irradiance, a major energy input for *Cyanidioschyzon* and we believe, to a lesser extent for *Galdieria*, both of which are the dominant primary producers in this environment (which had been observed by other studies at these sites [6–9]).

## Materials and methods

### Multi-omics sampling

Samples were collected from three distinct environments in Lemonade Creek, hereafter referred to as “Creek biofilm” (dense lush green biofilms in the creek channel), “Soil” (bordering the creek channel), and “Endolithic” (biomass within rocks sampled within centimeters of the creek flow). Each environment was sampled at a single time point in quadruplicate (n = 4) for Illumina short read metagenome data generation. Restrictions limiting the amount of material collected from the Endolithic environment meant that samples for metatranscriptomic (Illumina short read) and metabolomic (polar LC–MS) analysis were only collected from the creek biofilm and soil environments. These environments were sampled (n = 4) at four time points (TP1, commencing at sunrise, 07:30; TP2, ~ midday 12:50; TP3, early dusk, 16:50, and TP4, complete darkness, 19:25). Detailed protocols for sample collection, extraction, and data generation are presented in the Supplemental Information.

### MAG generation

In a previous publication [18], we used the metagenome assemblies described below to analyse viral sequences in the three Lemonade Creek environments. These data were included in the mapping analysis described below to enable accurate placement of reads.

For each sample, short read metagenome data was processed and assembled by the DOE Joint Genome Institute (JGI) following SOPs 1007.3 – LC, 1082.2, and 1077. This produced 12 metagenome assemblies (4 per environment), which had scaffolds from Cyanidiophyceae removed by a BLASTN comparison (>90% identity and > 90% coverage) against the available reference genomes. This analysis also elucidated that there were two Cyanidiophyceae species present at these sites, a *Cyanidioschyzon merolae* 10D-like isolate and a *Galdieria yellowstonensis* 5587.1-like isolate [previously *G. sulphuraria* [3]]. Prokaryotic MAGs were constructed from the Cyanidiophyceae cleaned assemblies using a comprehensive workflow, which combined multiple binning approaches, contamination assessment and cleaning, as well as per-environment reassembly and binning to aid the construction of MAGs from rare microbes (see Supplemental Information for details). The prokaryotic MAGs from the 12 samples and three per-environment re-assemblies were merged into a final non-redundant set of MAGs using an average nucleotide identity of 95% [widely assumed to be approximately equal to species]. Non-Cyanidiophyceae eukaryotic MAGs were constructed from the three per-environment reassemblies, although all five were identified in the Soil reassembly since it had the highest species diversity and thus produced the most contiguous assembly. Significant effort was put into ensuring these eukaryotic MAGs were free from prokaryotic contamination and had genes predicted using a taxa appropriate approach (i.e. ciliates, which we had a single MAG from, have unusual genome structure and non-standard codons which require special considerations; see Supplemental Information). The abundance of the non-redundant prokaryotic MAGs, the nuclear and organellar *C. merolae* 10D and *G. yellowstonensis* YNP5587.1 reference genomes, eukaryotic MAGs, and viral vOTUs (hereinafter, combined MAG dataset) assembled from the same samples in a previous analysis [18], were quantified across the 12 metagenome samples (encompassing the three environments) using bbmap v38.87 [19] and CoverM v0.6.1. Unbinned viral vOTUs were included in the mapping analysis to enable accurate placement of reads, however, they were not considered in the CoverM analysis since they were extensively analyzed in our previous publication [18].

### Gene expression analysis

For each sample, short read Poly-A and RiboMinus metatranscriptome data were sequenced and processed by the JGI following SOPs 1027.3, 1082.2, and 1077 (see Supplemental Information). Expression quantification was performed using salmon v1.8.0 [20] by mapping the processed Poly-A and RiboMinus reads (independently) against the genes predicted from the combined MAG dataset. The transcripts per million (TPM) values produced by Salmon were used for the full community gene expression analysis. To account for the affects that changes in community composition have on the relative gene expression results when analyzing individual species, the Salmon read counts for genes from *C. merolae* and *G. yellowstonensis* were extracted independently, with their TPM values recalculated using just these genes. This ensured that when we independently analysed each species' nuclear and organellar gene expression patterns, that the metrics for relative expression analysis (such as TPM, which quantifies expression of a gene relative to all other genes in the target set) are calculated specifically for the change in gene expression within the target species, and not on the change in expression due to shifts in community composition. That is, this analysis will reduce the effects that the presence or absence of other species has on the calculation of relative expression measures when considering the change in gene expression within a single species.

Orthogroup analysis, conducted using Orthofinder v2.5.4 [21], was used to identify the arsenic and mercury detoxification pathway genes in the combined MAG dataset. A parameter sweep was used to identify the optimal inflation value (3.0) for this analysis; using the known Cyanidiophyceae detoxification genes as standards for assessing gene family membership (see Supplemental Information). The constructed arsenic and mercury detoxification gene orthogroups had their TPM expression values from the full community expression results analysed to assess community wide detoxification, enabling the identification of taxonomic groups which express steps in these pathways at higher levels than expected based on the distribution of these genes across the MAGs.

### Metabolomics analysis

Samples were processed for targeted and untargeted polar metabolomics (positive and negative ionization modes) by the JGI using an integrated extraction and analysis workflow (see Supplemental Information). Differentially accumulated metabolites (DAMs) were identified using a two-sided *t*-test, as implemented in the *t.test* function from the stats v4.1.2 R package, with Benjamini & Hochberg adjusted *p*-values computed using the *p.adjust* function (method = “BH”) from the stats v4.1.2 R package.

### Protein structure analysis

Protein structures of ArsM (PDB: 4RSR) and ArsH (PDB: 2q62, just chain A) were downloaded from the Protein Data Bank and visualized in R v4.3.1 using the r3dmol v0.2.0 (https://github.com/swsoyee/r3dmol) package. Key active site residues were identified in the proteins of *G. yellowstonensis* 5572, MtSh, and *G. partita* SAG21.92 homologs and compared among homologs.

## Results

### Analysis of metagenome data from Lemonade Creek

The metagenome read data were mapped against the reference prokaryotic, eukaryotic, and viral MAGs, and the reference nuclear and organelle genomes of *C. merolae* 10D and *G. yellowstonensis* 5587.1 (Supplemental material). *C. merolae* was the single most dominant species in all three habitats (Figs. 1B), with its plastid genome comprising >50% of the TPM normalized data in the Creek biofilm and > 15% in the Endolithic and Soil samples (Figs. 1C). There was also a significant contribution by *G. yellowstonensis* in the Endolithic and Soil environments, as well as an increase in the proportion of reads derived from other eukaryotic MAGs in the Soil samples (Supplemental material). Beyond the algae, a modest contribution was made by bacteria, with <25% of reads being unassigned (i.e. do not map sufficiently well to the assembled reference genomes/MAGs to be classifiable; Table S1). The endolithic and soil habitats housed a relatively more complex biotic assemblage. More reads derived from Archaea were present in the Endolithic samples, with more bacterial derived reads in both the Endolithic and Soil samples.

The distribution of algae and prokaryotes in Lemonade Creek was studied using sequence co-abundance (Fig. S1, Supplemental material) and single-nucleotide polymorphism (SNP) data (Fig. 1D). These approaches provided consistent results. The SNP analysis showed that both the local *C. merolae* and *G. yellowstonensis* Soil populations have a high degree of distinctness in principal component (PC) 1. Furthermore, whereas the *C. merolae* Creek biofilm and Endolithic populations show a relatively low fixation index (F_ST_) and shared peak regions, the *C. merolae* Soil population had an overall elevated F_ST_ as well as unique peaks, indicating these are distinct populations relative to the other sites (Figs S2–S4), perhaps reflecting species differentiation. Similar trends were observed with the *G. yellowstonensis* populations, although the existence of fewer SNPs in the Creek biofilm population made these calculations less robust (Figs. S5 and S6).

### Gene expression pattern of YNP MAGs

**Figure 2:**
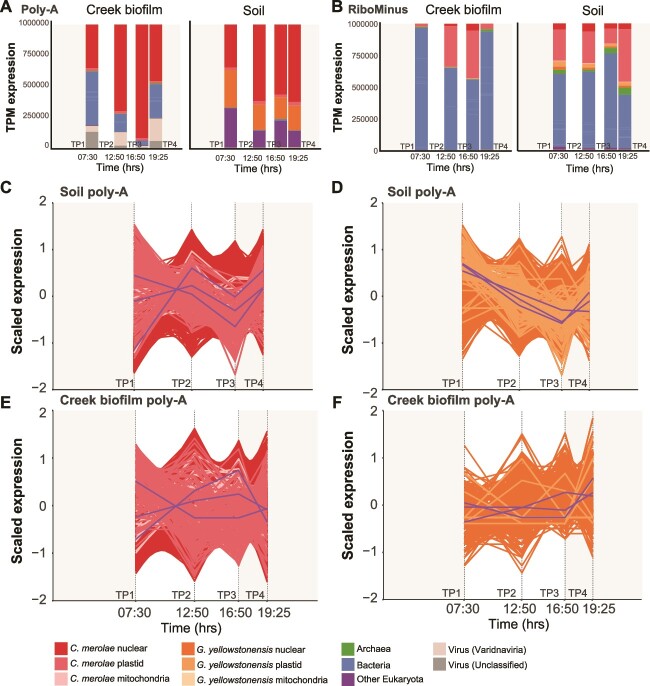
Analysis of YNP metatranscriptome data. Relative abundance (TPM) of (A) Poly-A and (B) RiboMinus metatranscriptome reads mapped against the predicted genes from the assembled YNP metagenome data and reference algal genomes (see legend). Each bar in the graph represents the average value of the n = 3 replicates per time point that were analyzed. Expression patterns of all *C. Merolae* 10D (C) and *G. Yellowstonensis* 5587.1 (D) genes in the Soil Poly-A libraries. The patterns are presented as log_2_ z-score normalized values. Each point in the line graph represents the average value of the n = 4 replicates per time point that were analyzed. The purple lines in the *C. Merolae* 10D (C) and *G. Yellowstonensis* 5587.1 (D) panels represent the average expression value of all transcripts at each time point; the three purple lines in each plot represent the average expression of nuclear, plastid, and mitochondrial genes. (E, F). The same analysis of expression patterns done for the Creek biofilm samples.

Poly-A (eukaryote) and RiboMinus (total RNA) metatranscriptomic reads from the Creek biofilm and Soil samples were mapped against coding sequences from the non-redundant YNP MAGs and the *C. merolae* 10D and *G. yellowstonensis* 5587.1 reference nuclear and organelle genomes to determine relative gene expression levels. Given the observed biotic complexity of the samples and the vastly different abundances of taxa across them, these data are best interpreted as community level shifts in transcription rather than absolute quantification of gene expression in a particular MAG. In the Creek biofilm Poly-A transcript data, many reads mapped to bacterial CDS, likely due to contamination [although some bacterial mRNAs are polyadenylated [22]], which resulted in bacteria comprising a significant fraction (from 1.2% at TP3 to 49.2% at TP1; Table S2) of the expressed transcripts (measured as TPM; Fig. 2A, left image). As expected, *C. merolae* was the single largest (per genome) contributor to Poly-A transcripts (22%–96%; Table S2), as expected, based on the metagenome data. The Soil Poly-A transcript data did not contain a significant number of prokaryotic transcripts (Fig. 2A, right image), and *C. merolae* was again dominant in gene expression. Other eukaryotes comprised a significant fraction of the expressed transcripts, with the amoeba *Acanthamoeba* sp. totaling between 3.3% and 23% of the expressed transcripts at each time point (Fig. 2A right image; Table S2). In the Creek biofilm RiboMinus data, the majority (55.4%–96.3%) of transcripts were bacterial derived (Fig. 2B, left image; Table S3) and the large increase in the relative abundance of *C. merolae* nuclear and organellar transcripts at TP2 (34.4%) and TP3 (43.7%) compared to TP1 (2.4%) and TP4 (4.3%) was driven completely by plastid genes. In the Soil RiboMinus data, most transcripts are bacterial derived (42.4–74.4%), however, there was an increase in the proportion of archaeal (1.7%–5.8%), *G. yellowstonensis* (3%–6.6%), and other eukaryote (0.97%–2.4%) derived transcriptomes across all time points (Fig. 2B, right image; Table S3). As found for the Creek biofilm data, there was a large contribution to the overall transcript pool by *C. merolae* plastid genes (11.9%–41.5%).

These data demonstrate that *C. merolae* and many other organisms present in Lemonade Creek follow a diurnal cycle. In the *C. merolae* Soil samples, 93% of nuclear genes in the Poly-A transcript libraries increased significantly (|FC| > 1 and adjusted *P*-value <0.05) in their relative abundance between TP1 and TP2 (Fig. 2C); however, there was a noticeable dip at TP3, before an increase at TP4. *G. yellowstonensis* showed a clear trend of decreasing relative abundance from TP1 to TP3, with an increase at TP4 (Fig. 2D). There was a sharper dip at TP3, when *C. merolae* also showed a decrease. This may result from a significant increase in prokaryotic transcription at TP3, or an overall drop in *C. merolae* plastid gene expression coinciding with significantly decreased light levels (Fig. S7A), which constitutes a significant proportion of the RiboMinus data (Fig. 2B). In the Creek biofilm, 96.1% of *C. merolae* nuclear predicted genes increased significantly in their relative transcript abundance between TP1 and TP2, with none being significantly reduced (Fig. 2E). Of the 6004 *G. yellowstonensis* nuclear genes, 80 (1.3%) significantly increased and none were significantly decreased (Fig. 2F). Because *G. yellowstonensis* comprises a minor proportion of the Creek biofilm metagenome and metatranscriptome samples, the low number of differentially abundant genes in this species is unsurprising.

RiboMinus reads mapped to *C. merolae* genes were extracted and TPM values recalculated to remove the influence that changes in community composition has on the TPM results. These recalculated results show that most expressed *C. merolae* transcripts at each time point are plastid derived (Fig. S7B and S7C), with the photosystem II D1 (Q[b]) protein (*psbA*) constituting 38.9%–66.5% of transcripts in the Creek biofilm (Fig. S7B) and 18%–45% in the Soil (Fig. S7C). In addition, antenna proteins (phycocyanin alpha and beta chain [*cpcA* and *cpcB*] and allophycocyanin alpha and beta chains [*apcA* and *apcB*]) constitute between 7.4% and 16.2% of total expressed transcripts in the Creek biofilm and 15.3%–21.8% in the Soil. In the case of *G. yellowstonensis*, *psbA* constitutes 3.5%–19.7% of Creek biofilm and 13.6%–21% of Soil transcripts, and antenna proteins constitute 20.2%–26.3% of Creek biofilm and 20.7%–22.7% of Soil transcripts. There are also several nuclear *G. yellowstonensis* genes with high abundance (>1% total transcripts) in the RiboMinus data, suggesting that this species may be relying more on heterotrophy for survival, compared to *C. merolae*, which shows high plastid gene expression.

### Arsenic and mercury detoxification in Lemonade Creek

**Figure 3:**
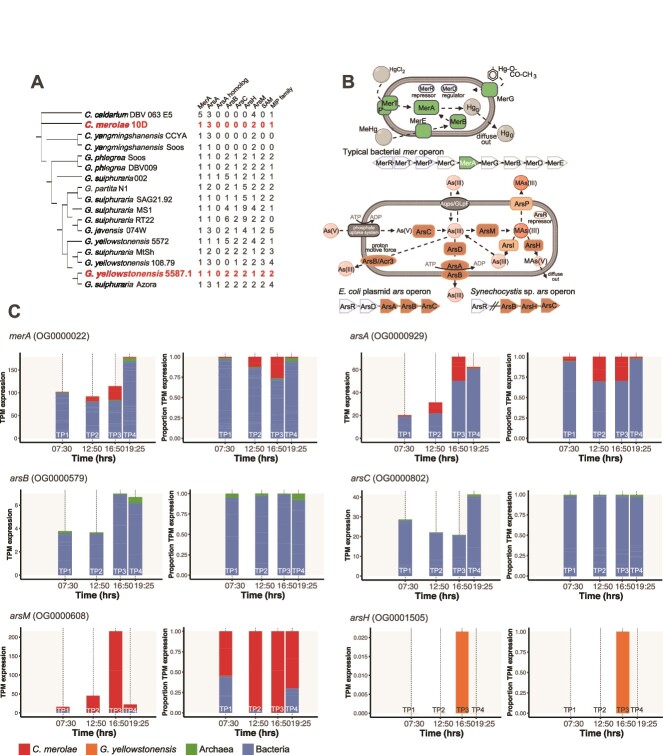
Analysis of *mer* and *ars* genes. (A) Distribution and copy number of *mer* and *ars* genes in a canonical phylogeny [3] of Cyanidiophyceae. The species (or closely related strains) present in the studied YNP habitats are shown in the red text. (B) Schematic prokaryotic cells showing canonical mercury (top) and arsenic (bottom) detoxification pathways. The cells portray the relevant enzymes, however, not all prokaryotes encode all components of these pathways. (C) Contribution of taxonomic groups to the arsenic and mercury detoxification pathways in the Creek biofilm samples. The TPM expression values of all genes from an orthogroup identified as containing genes putatively from a specific step in the detoxification pathway is shown as a stacked bar graph (left). The proportion of TPM values contributed by each taxonomic group is shown as a stacked bar graph (right).

Analysis of the diurnal RiboMinus RNA data showed that the relative accumulation pattern of *merA* (OG0000022) in both habitats is dominated by bacteria, with only a minor contribution from *C. merolae* (Figs. 3C and S8). The transcript accumulation pattern of *ars* genes is more complex with the *arsC* OG (OG0000802) predominantly expressed by bacteria in the Creek biofilm, and by bacteria and *G. yellowstonensis* in the Soil. The potential for As(III) export was more difficult to interpret using our data. Bacterial and *C. merolae arsA* (OG0000929) peaked in abundance at TP2 and TP3 in the Creek biofilm, whereas *arsB* (OG0000579) was dominated by bacterial taxa with the similar diurnal pattern as *arsA* (these genes are likely to be co-localized in an operon and therefore, co-regulated). The Soil samples were more algae-driven, with *G. yellowstonensis* and *C. merolae* OGs dominating the *arsA* and “arsenic transporter” accumulation patterns. *G. yellowstonensis* and bacteria dominated *arsB* expression (Fig. S8). The detected transcripts of *arsM* (OG0000608) in the Creek biofilm were derived almost entirely from *C. merolae* (excluding a small bacterial contribution at TP1 and TP4). Furthermore, the change in accumulation of this *C. merolae* OG was relatively broad over the four time points, ranging from <20 TPM at TP1, to >200 at TP3, and < 50 TPM at TP4. The Soil samples showed a similar pattern, however, significant contributions were also made by *G. yellowstonensis* and other eukaryotes (Fig. S8). Prokaryotes comprised a minor (< 20% total expression) fraction of the detected *arsM* transcripts. Finally, *arsH* (OG0001505) expression in the Creek biofilm was nearly undetectable in the RiboMinus data, with only minor contributions (~0.02 TPM) from *G. yellowstonensis* at TP3. In Soil, *G. yellowstonensis arsH* was more strongly accumulated, peaking at TP1, and then declining over the day (Fig. S8).

### Protein structure

**Figure 4:**
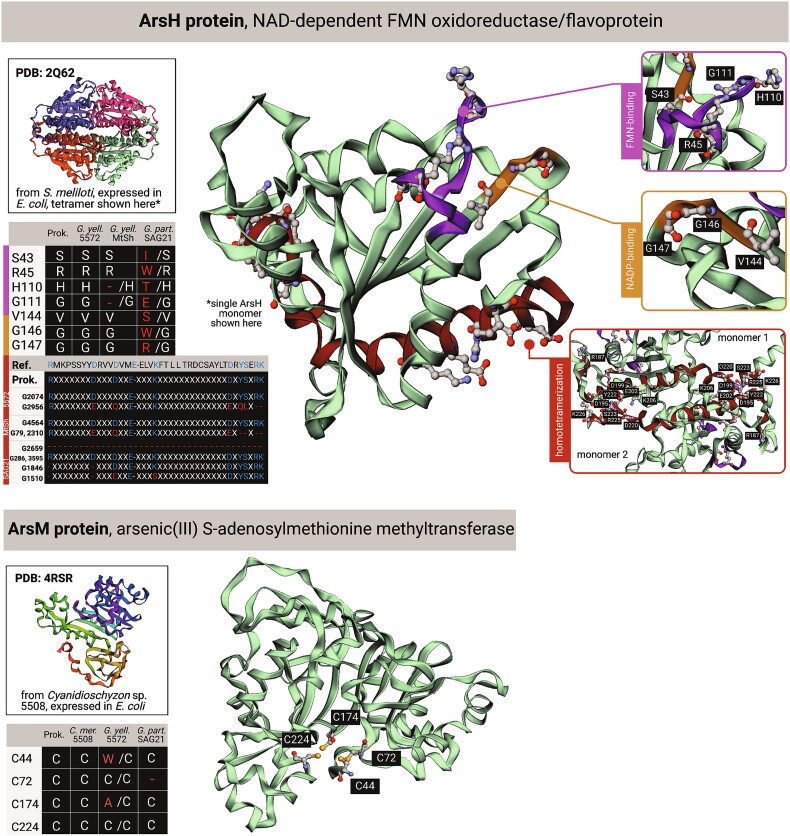
Loss of conserved residues in Cyanidiophyceae ArsH and ArsM. The reference bacterial structures are shown for *arsH* and *arsM*, as are alignments and locations of conserved site changes in the structures that are present in the sequences of the proteins in *G. yellowstonensis* 5572, MtSh, and *G. partita* SAG21.92. These data suggest that the algal *ars* gene original functions may be compromised or lost (supplemental material). Made in biorender.

Using existing bacterial protein structures as reference, we analysed *ars* genes in Cyanidiophyceae to determine whether their functions may potentially have been altered, compromised, or lost. For ArsH (beyond *G. partita* SAG21.92), key residues involved in FMN-binding and homotetramerization have been lost (highlighted in Fig. 4 top). At least one of four ArsM proteins in *G. yellowstonensis* 5572 showed evidence of loss of function; G1662 retains only two of the four conserved cysteines involved in As(III)-binding [23] and also lacks most of the residues involved in S-adenosylmethionine (SAM)-binding [24] (highlighted in Fig. 4 bottom).

### Cell cycle and the diurnal phototrophy–heterotrophy cycle

Light responses, the cell cycle, and metabolism of *C. merolae* and *G. yellowstonensis* in the Lemonade Creek habitats were evaluated using the Poly-A expression pattern of target genes (Table S4). *G. yellowstonensis* was evaluated based solely on Soil data because of the paucity of reads from the Creek biofilm samples (Fig. 2A). In *C. merolae*, two copies of the cryptochrome DASH gene, a subclade of the cryptochrome/photolyase family, some of which accumulate at midday in other organisms [25], exhibited a diurnal pattern, peaking at midday in the Creek biofilm samples (Fig. S9A). In *C. merolae*, as observed in lab cultures over the diurnal cycle (12-hour light/12-hour dark) [26], genes involved in glycolysis/gluconeogenesis (e.g., 6-phosphofructokinase; Fig. S9C), the entrance to the TCA cycle (e.g., dihydrolipoamide dehydrogenase; E3 component of pyruvate dehydrogenase complex; Fig. S9E), and the antenna of the photosystem (chlorophyll *a* binding protein; Fig. S9G) were upregulated during the day and downregulated at night. Conversely, cell division genes (S- and M-phase cyclins and the chloroplast division gene *ftsZ*) and lactate fermentation (L-lactate dehydrogenase) were upregulated during the night in the Creek biofilm samples (Fig. S9E). These results suggest that, in *C. merolae*, as observed in synchronized lab cultures [27], in the creek environment where this alga coexists with other microorganisms, the cells exhibit higher respiratory activity due to photosynthesis during the day but lower respiratory activity at night, with an increased reliance on fermentation for ATP production. However, in the Soil samples, these diurnal rhythms were not evident in *C. merolae* and *G. yellowstonensis* (Fig. S9). We postulate that this is explained by the cells in the soil being exposed to lower light levels during the day and due to the mixotrophic lifestyle of *G. yellowstonensis*.

## Discussion

### *C. merolae* dominates the Lemonade Creek biofilm environment

The canonical *C. merolae* that has been extensively studied in culture is a haploid, dumbbell-shaped cell that lacks a cell wall and is an obligate photoautotroph [28]. This species has a highly reduced genome of size 16.5 Mbp (4803 nuclear protein coding genes) and is considered to be a specialist, limited to aqueous environments [28]. However, it is possible that a diploid cell-walled life cycle stage may exist for this species in nature, as has recently been demonstrated for diploid *Galdieria* which have a sexual cycle that includes a cell wall-less haploid stage [29]. Regardless of which life cycle stage (or mixture) inhabits the Lemonade Creek environments we have studied, the dominance of this species [also noted by other studies at these sites [6–9]] suggests that *C. merolae* outcompetes the physiologically more flexible local *Galdieria* (*G. yellowstonensis*) populations. *Galdieria* is renowned for being able to utilize over 50 sources of carbon for energy [30]. These data also provide a potential explanation for the larger *Galdieria* gene inventory (e.g., 6004 nuclear protein-coding genes in *G. yellowstonensis* 5587.1) and their heterotrophic capacity. Namely, these algae retain the ability to use different carbon sources, which may avoid them competing directly with *C. merolae*. SNP analysis demonstrates that despite their close proximity (i.e. within cm), there is significant spatial partitioning of Lemonade Creek biota, which we have previously demonstrated with viruses at this geothermal feature [18].

The complexity of the microbial community in these environments and the relative nature of RNA-seq data, make it challenging to disentangle differential regulation of genes in a particular organism, and the overall shift in the composition of the community. That is, the apparent increase in gene expression in *C. merolae* could be a result of true upregulation of gene expression in this species, the downregulation of genes in other organisms in the community, or a mixture of both processes (the latter being more likely). Regardless of the mechanism, the prokaryote and eukaryote species in these communities clearly follow a diurnal cycle. This result is confirmed by the large contribution of *C. merolae* plastid genes to the RiboMinus transcriptome pool in both environments (Fig. 2B). The clear abundance of *C. merolae* plastid genes (particularly the *psbA* and the antenna proteins, which often comprised most of the expressed transcripts) in both the total and species-specific expression results (Figs 2B, S7B, S7C) and lack of other major photosynthetic taxa in the sequence data demonstrate that it is the dominant primary producer in both the Creek biofilm and Soil habitats. In contrast, the mixotrophic *G. yellowstonensis* has a lower proportion of its transcripts assigned to plastid-derived genes (particularly, *psbA*; Figs. S7D, S7E), suggesting that it likely has a higher reliance on its heterotrophic capabilities to scavenge from the environment, rather than living as a pure autotroph. This pattern is supported by the untargeted and targeted metabolomic data (Supplemental material), which show that the largest number of DAMs in the Creek biofilm is between TP1 and TP2 (Table S5), consistent with strong, light-driven diurnal cycling in these habitats. It also provides insights into the complex biochemical interactions at these sites which supports the long term microbial stability of these environments [31].

The pattern of gene expression we observe is consistent with previous lab-based work with synchronized *C. merolae* 10D cultures over the diurnal cycle that show photosynthetic activity to peak at midday. Cell cycle progression (G1-S transition) occurs at night to minimize DNA damage caused by redox stress [26, 30]. The latter aspect is critical at the study site where TP2 (midday) light levels (2003 mmol m^−2^ s^−1^) at Lemonade Creek were one to two orders of magnitude greater than the other times when samples were collected on October 10, 2021 (Fig. S7A). However, actual levels in the creek biofilm and soil were likely lower due to self-shading. For more details, see Supplemental material.

### Arsenic and mercury detoxification pathways are split across multiple taxonomic domains

The geothermal springs in YNP contain significant levels of mercury [27, 32], particularly in acidic features [10], including Lemonade Creek [33]. Acidic environments favor the occurrence of Hg^2+^ [32] suggesting that low pH may have influenced the evolution of *merA* in YNP. A single copy of this detoxification gene is present in both *G. yellowstonensis* and *C. merolae*. In contrast to *merA*, most *ars* genes are present in multiple copies (Fig. 3A). MerA reduces Hg^2+^ to volatile Hg^0^ that passively leaves the cell [34] (Fig. 3B), providing resistance. These genes originated from bacteria *via* HGT and share high sequence similarity to prokaryotic MerA, retaining the conserved cysteine residues essential for activity [35]*.* Consistent with this idea, MerA in *G. yellowstonensis* 5572 and *G. partita* SAG21.92 (inferred using gene expression analysis) rapidly detoxifies mercury in unialgal culture [36]. The expression of *merA* in the Creek biofilm and Soil habitats is dominated by bacteria, with only a minor contribution from *C. merolae* (Figs. 3C, S8).

Both *G. yellowstonensis* 5587.1 and *C. merolae* 10D encode various *ars* genes, yet the compositions (Fig. 3A) and encoded functions differ (see below). And in contrast to *merA* (above), most *ars* genes in these algae are present in multiple copies, all of which were acquired in Cyanidiophyceae *via* HGT (Fig. 3A). In the Creek biofilm, based on *arsC* expression levels, As(V) to As(III) conversion by ArsC is primarily a bacterial function, decreasing slightly at TP2 and TP3, and possibly interpreted as shifts in community composition over the diurnal cycle. *C. merolae arsM* expression (encodes for methylation of As(III)) peaks at TP3, which is when this species is most photosynthetically active (inferred using plastid gene expression data; Fig. 2B) MAs(III) to MAs(V) conversion, as inferred by *arsH* expression, appears to be absent in this environment. This could be explained by trimethylarsine gas production by ArsM and its spontaneous oxidation to trimethyarsine oxide (TMAsO) [37], which is essentially inert, obviating the need for ArsH. The As(III) efflux genes are predominantly expressed by bacteria, with only the ArsA/ATPase family being expressed at TP2 and TP3 by *C. merolae* [38]. It is worth noting that the *arsC* and *arsM* gene families are broadly distributed across MAGs in our analysis, thus, their expression above detectable levels by specific groups in this environment clearly demonstrates the partitioning of this detoxification pathway across taxonomic domains. The *arsH* gene family is not very broadly distributed, suggesting that it is not an essential part of the arsenic detoxification pathway in the Creek biofilm, apparent in the low relative gene abundance values. The more biotically diverse soil habitat follows a similar trend, however, the Cyanidiophyceae are more dominant contributors to arsenic detoxification, with *C. merolae* again acting as the key methylator of As(III) (Fig. S8). Whereas the interaction of *ars* gene expression (and their protein products) across different lineages is inferred here, it is noteworthy that both habitats show internally consistent (albeit different) patterns for this pathway. In the Creek biofilm, all genes except *arsC* show peak relative accumulation at TP3 (early dusk), whereas in Soil, these patterns are more varied with several peaking at TP1 (at or before sunrise). The As(v) to MAs(V) metabolic pathway is a sequential process, requiring the *arsC*, *arsM*, and *arsH* genes. Whereas we do not know the exact type or amount of arsenic present in the creek at the time of sampling, previous studies have reported arsenite [As(III)] to be the most common at these locations [6, 7, 9, 13]. We therefore interpret the differential presence/absence and expression of these genes in bacteria, *C. merolae*, and *G. yellowstonensis* as demonstrating arsenic detoxification in these environments as a process that is performed through the interaction between multiple lineages. This process may involve the detoxification of arsenite [As(III)] to MAs(V) by *C. merolae* and *G. yellowstonensis*, or its reduction to arsenate by prokaryotes. These data suggest a complementary, community-wide arsenic detoxification response in both YNP habitats.

Given the extensive primary literature characterizing different *ars* genes, these sequences are ideal for testing the integrated HGT model (IHM) for eukaryotes [36]. The IHM posits that when conditions that initially favor HGT fixation diminish or disappear (e.g., due to toxin absence or the evolution of community detoxification) foreign gene(s) may survive if they are integrated into a broader stress (or other) responses that favor retention (Supplemental material). This hypothesis was built using gene co-expression data for the five *arsH* genes in *G. partita* SAG21.92 which showed them to be strongly linked to clusters of co-expressed genes encoding protein translation and photosynthesis [36]. The IHM contrasts with the standard model of HGT, as for *mer* genes, whereby this sequence has retained its original function for hundreds of millions of years [36]. Analysis of *ars* gene protein structure point to a potentially significant outcome of HGT and complex community interactions in eukaryotes: foreign genes may survive *via* duplication, divergence, and putative neofunctionalization that allows them to persist long-term. This is demonstrated by the loss of key functional residues in many *arsH* and *arsM* gene copies (Fig. 4).

## Conclusions

Geothermal habitats in Lemonade Creek, YNP were used to investigate microbial interactions and their impact on gene expression over the diurnal cycle and on longer-term genome evolution. Our work leads to four major insights: (i) the omics data demonstrate that Cyanidiophyceae are the primary drivers of life in these highly acidic geothermal habitats with *C. merolae* as the dominant phototroph (Figs. 1B, 2, 5). (ii) The cell and diurnal phototrophy-heterotrophy cycles are strongly shaped by local conditions, with shifts in species composition occurring at the scale of centimeters (Fig. 1D). (iii) Arsenic and mercury detoxification appear to be a “community affair” with members of the three domains of life sharing these duties (Fig. 3). *C. merolae* plays a pivotal role as the dominant producer of methylarsenicals that, if they remain as highly toxic trivalent species [e.g., MAs(III)], may regulate local biodiversity *via* microbial warfare across short distances. If, however, they are spontaneously oxidized to inert pentavalent species (e.g., TMAsO), this would play a key role in arsenic detoxification in Lemonade Creek. (iv) Community based arsenic detoxification has putatively allowed algal *ars* genes to undergo duplication and sequence divergence, potentially taking on novel functions (Fig. 4), as predicted by the IHM. This model is akin to the Black Queen Hypothesis, whereby community interactions (e.g., provision of public goods [metabolites]) can drive prokaryote genome reduction [39]. If the IHM applies more generally, then highly specialized genes derived *via* HGT and their duplicated copies, may have been the source of novel functions that supported the early evolution of eukaryotes.

## Supplementary Material

Supplementary_Figure_1_ycae151

Supplementary_Figure_2_ycae151

Supplementary_Figure_3_ycae151

Supplementary_Figure_4_ycae151

Supplementary_Figure_5_ycae151

Supplementary_Figure_6_ycae151

Supplementary_Figure_7_ycae151

Supplementary_Figure_8_ycae151

Supplementary_Figure_9_ycae151

Supplementary_Figure_10_ycae151

Supplementary_Figure_11_ycae151

Supplementary_Figure_12_ycae151

Supplementary_Figure_13_ycae151

Supplementary_Figure_14_ycae151

Stephens_etal_2024_Supplemental_Tables_1-21_ycae151

Stephens_etal_2024_ISME_Comm_SupplementaryInformation_ycae151

## Data Availability

All experimental data pertinent to this manuscript is accessible for review, either in the manuscript, in a public database, or as material uploaded with the manuscript as additional files for review purposes. The metagenome and metatranscriptome data associated with this project are available under the Umbrella BioProject link https://www.ncbi.nlm.nih.gov/bioproject/PRJNA1135266. The assembled non-redundant MAGs and their associated predicted genes and annotations are available from https://zenodo.org/records/14166326. Targeted and untargeted polar metabolomics results are available from https://zenodo.org/doi/10.5281/zenodo.12709950. Feature based molecular networking results for the metabolomics data are available at the Global Natural Products Social Molecular Networking (GNPS) site https://gnps.ucsd.edu/ProteoSAFe/status.jsp?task=8449ead7d6b94e208ceccee688909d83 for the positive mode data and at https://gnps.ucsd.edu/ProteoSAFe/status.jsp?task=013e5b18bc804c9ab5a0f494e297ca73 for the negative mode data. The raw metabolomics data are also available with the MassIVE accession number MSV000095232 and at https://massive.ucsd.edu/ProteoSAFe/dataset.jsp?task=7b95c3e1865444ebbc60d1316517d7f0.
