# Supplementary figures and images for: Temporal dynamics in a red alga dominated geothermal feature in Yellowstone National Park
Source: ISME Commun. 2024 Dec 3;4(1):ycae151. doi: 10.1093/ismeco/ycae151 (PMC11662350; doi:10.1093/ismeco/ycae151)

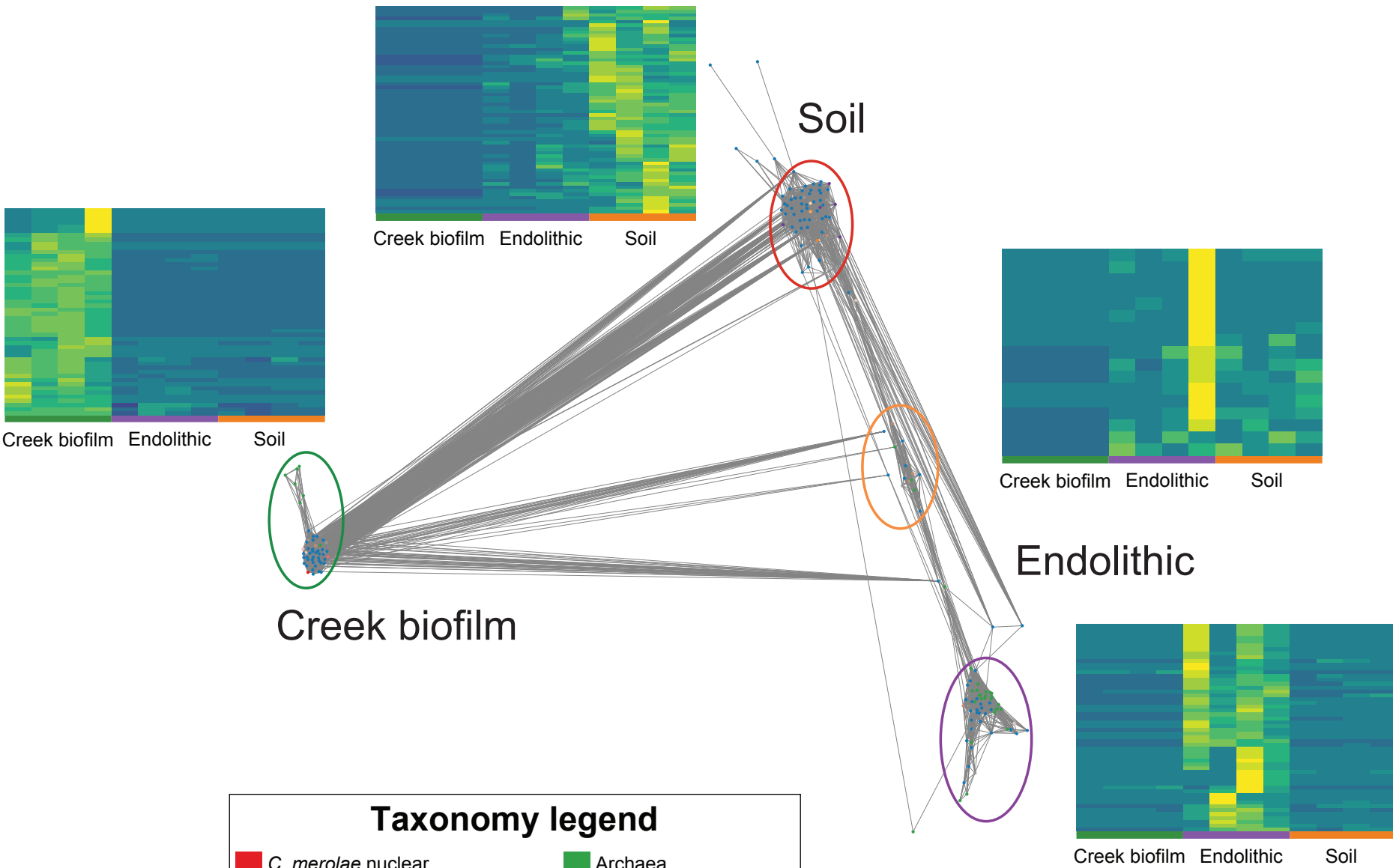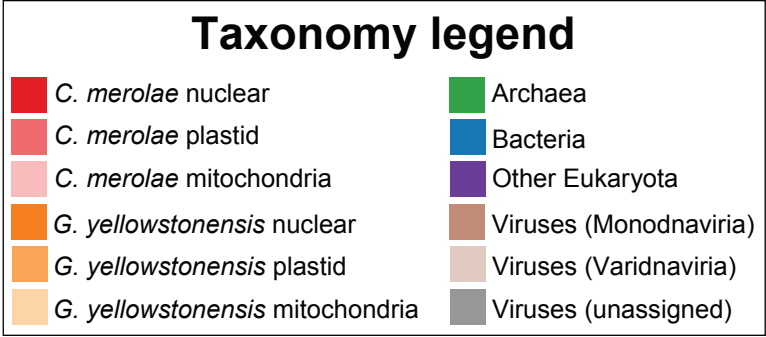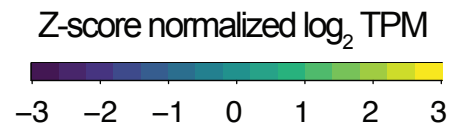

Supplement: Supplementary_Figure_1_ycae151 [file supplementary_figure_1_ycae151.pdf]

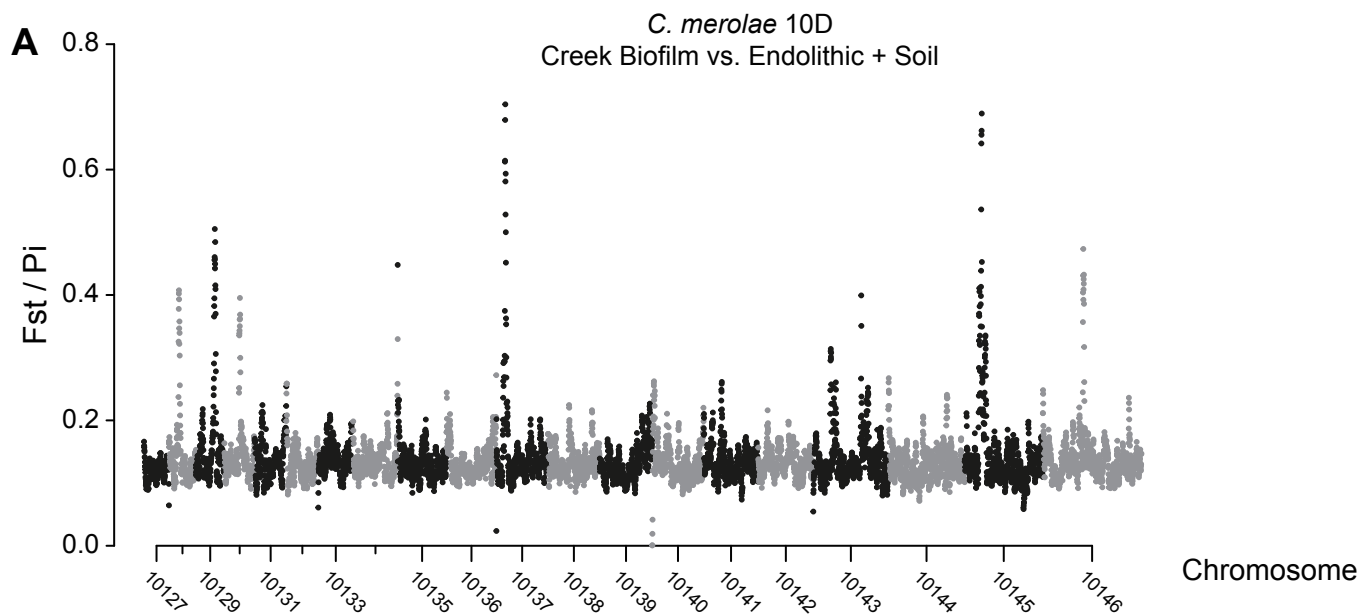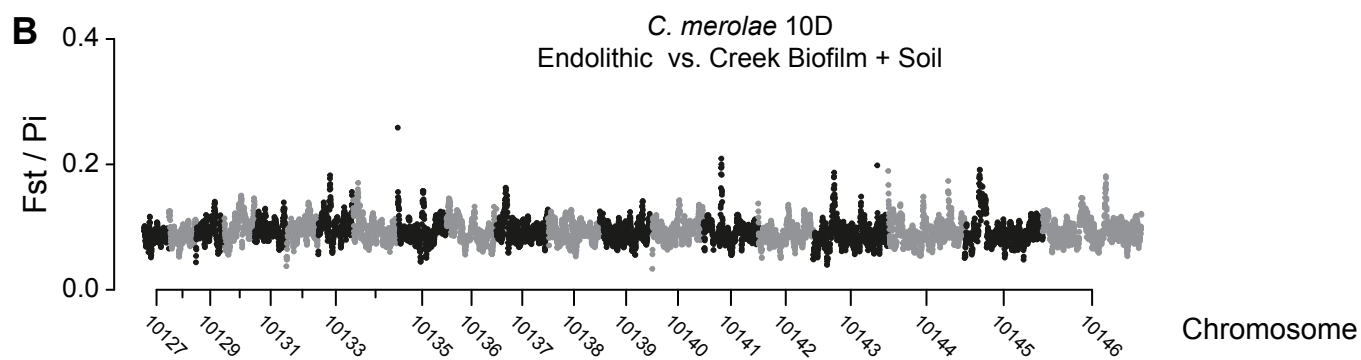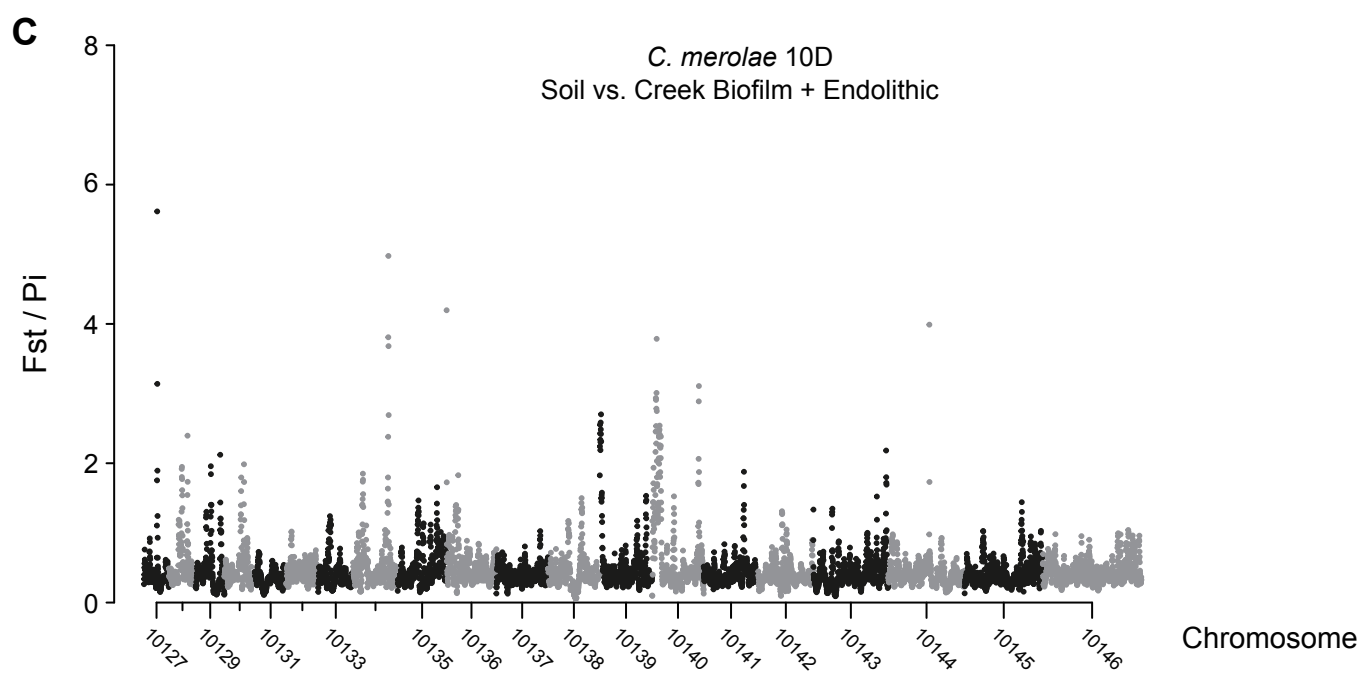

Supplement: Supplementary_Figure_2_ycae151 [file supplementary_figure_2_ycae151.pdf]

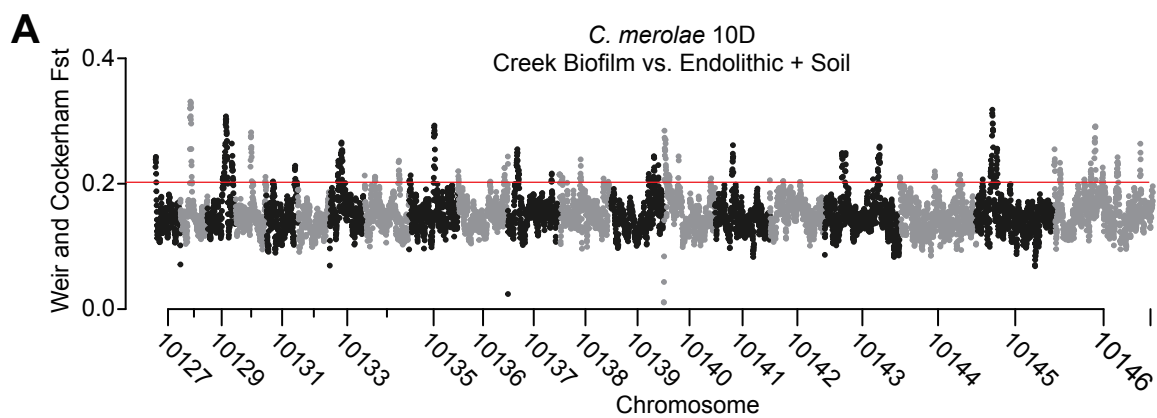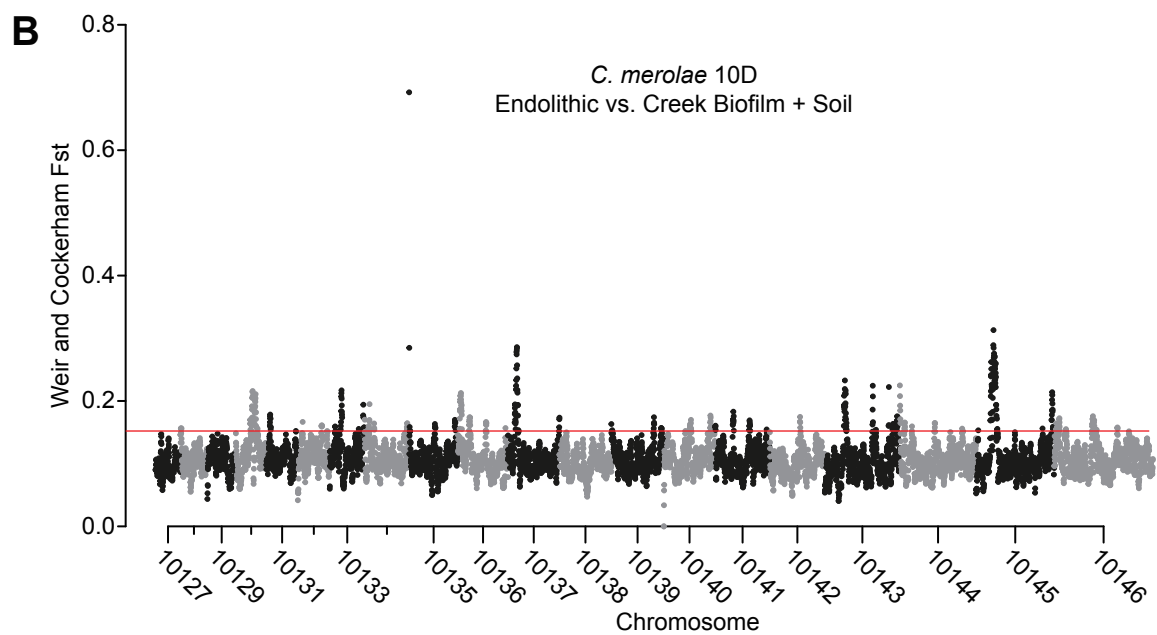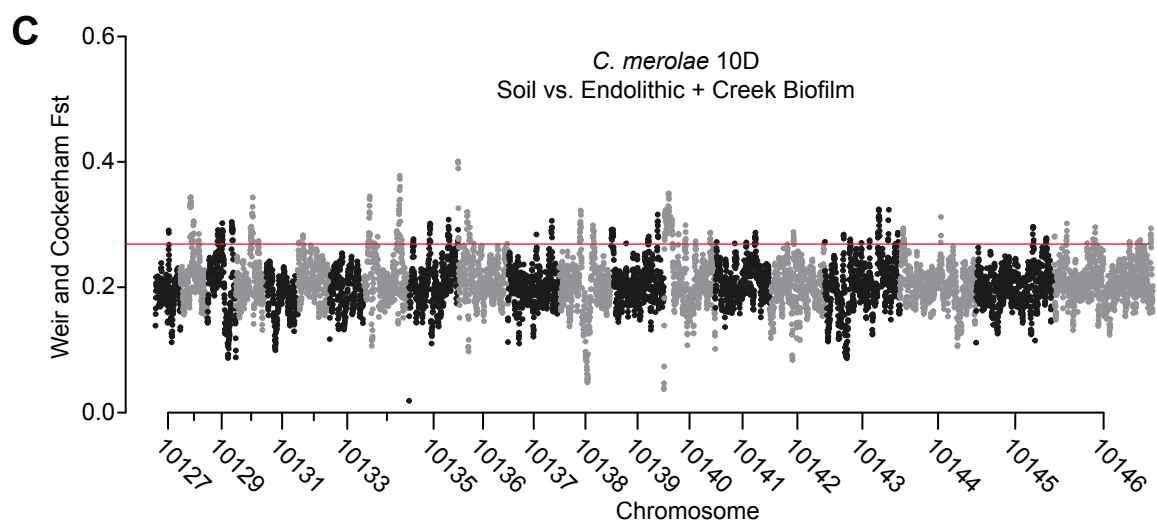

Supplement: Supplementary_Figure_3_ycae151 [file supplementary_figure_3_ycae151.pdf]

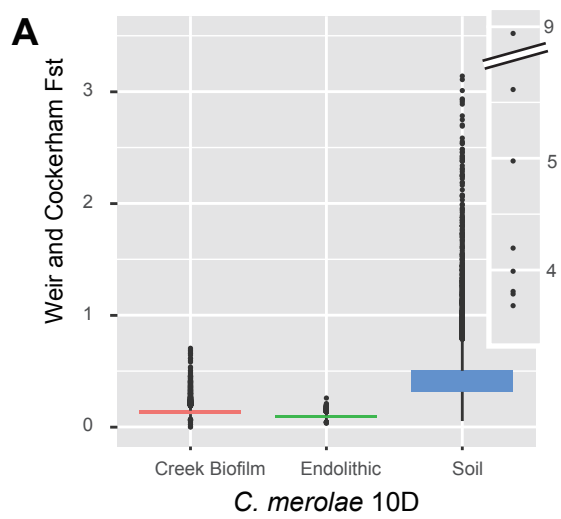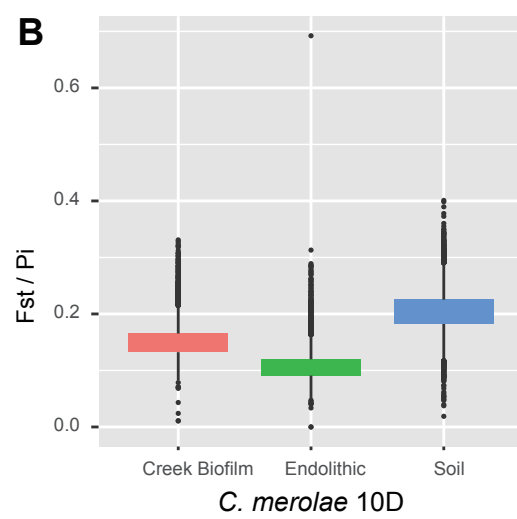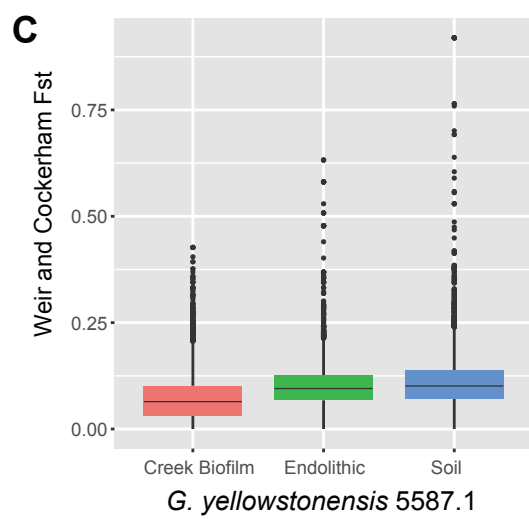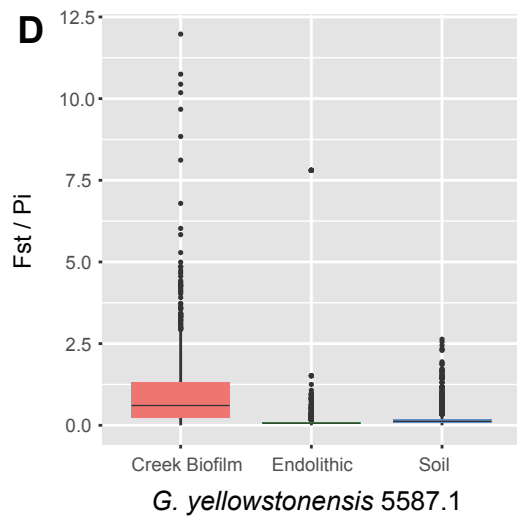

Supplement: Supplementary_Figure_4_ycae151 [file supplementary_figure_4_ycae151.pdf]

**A**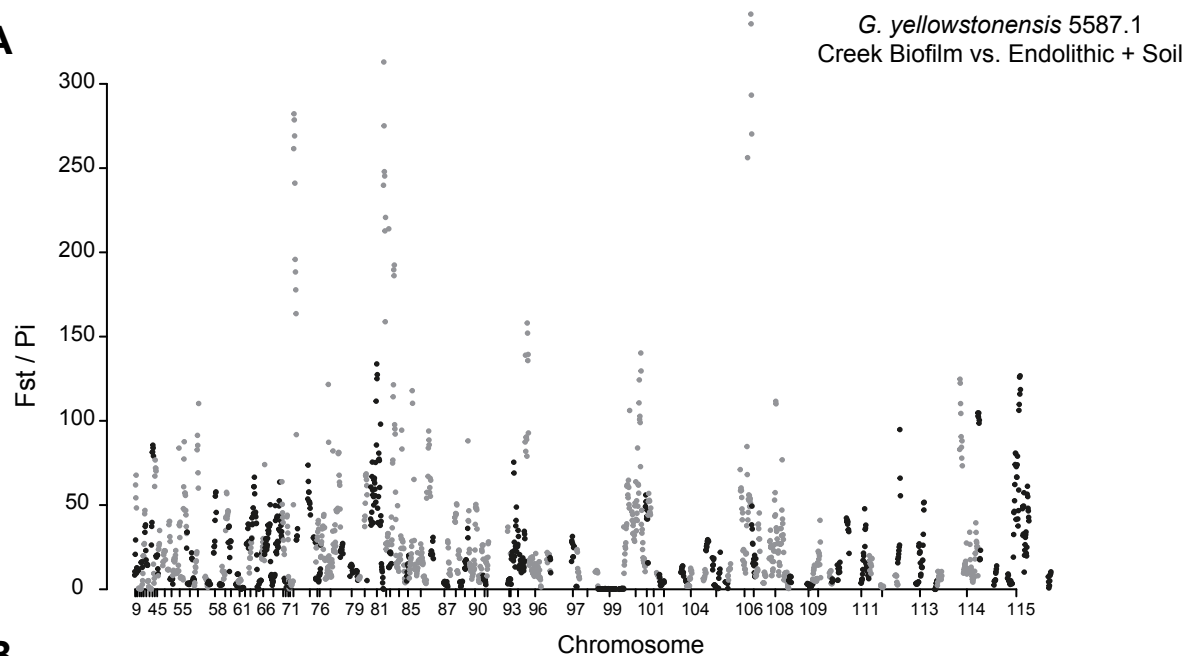**B**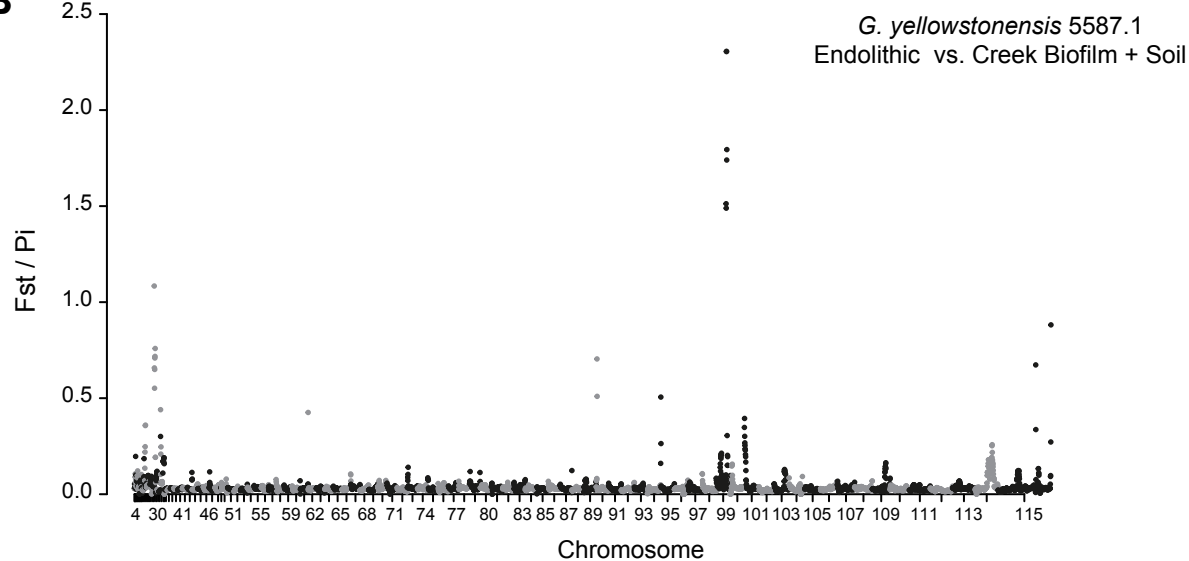**C**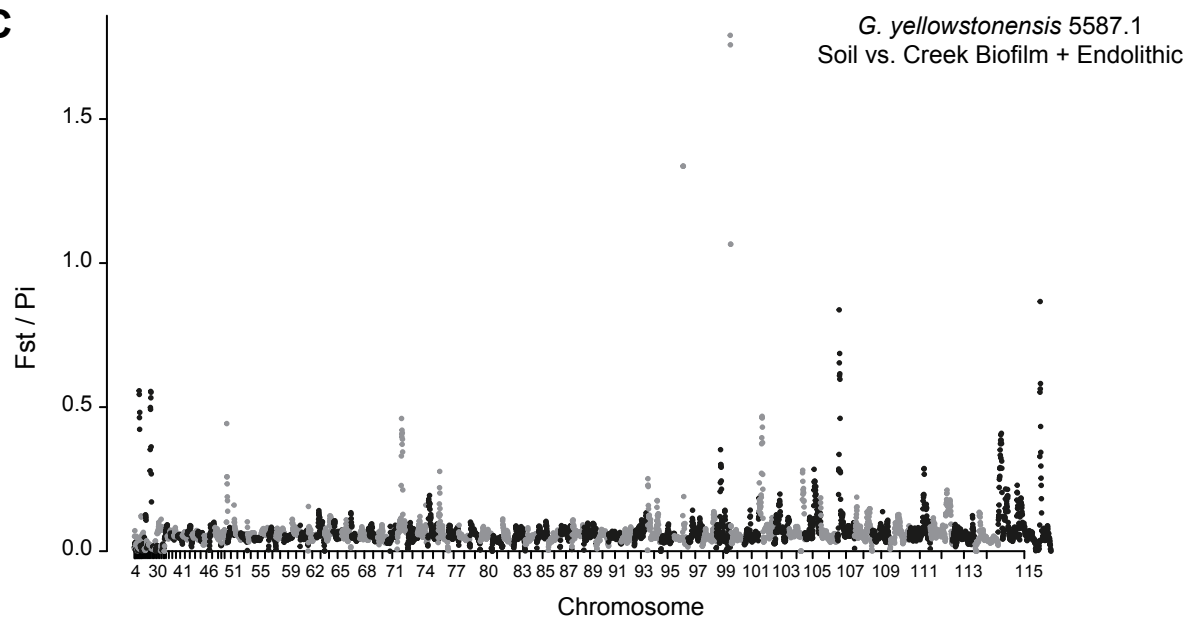

Supplement: Supplementary_Figure_5_ycae151 [file supplementary_figure_5_ycae151.pdf]

A

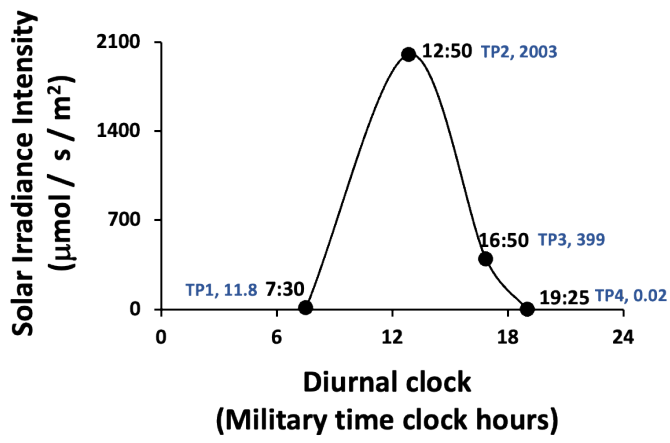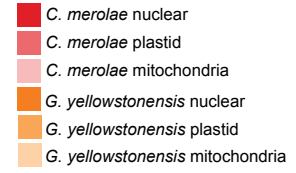

B

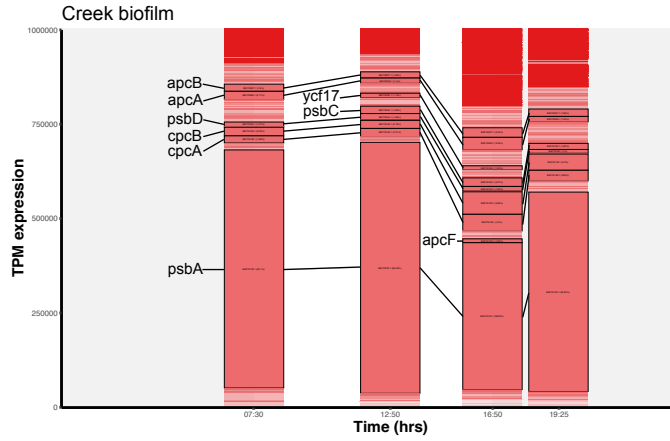

C

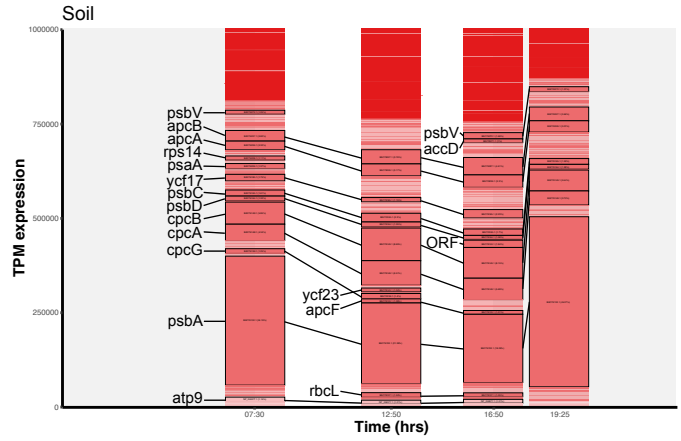

D

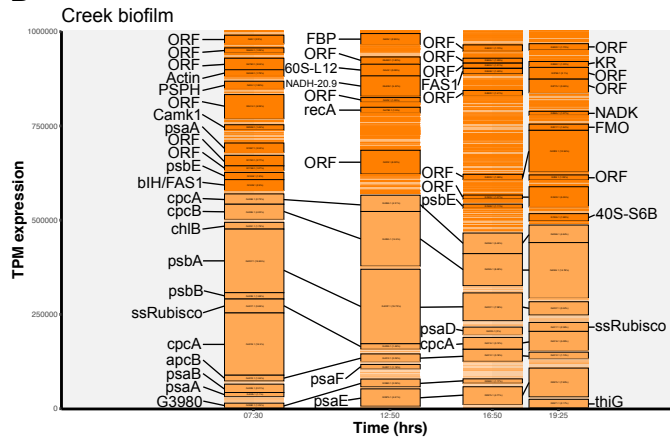

E

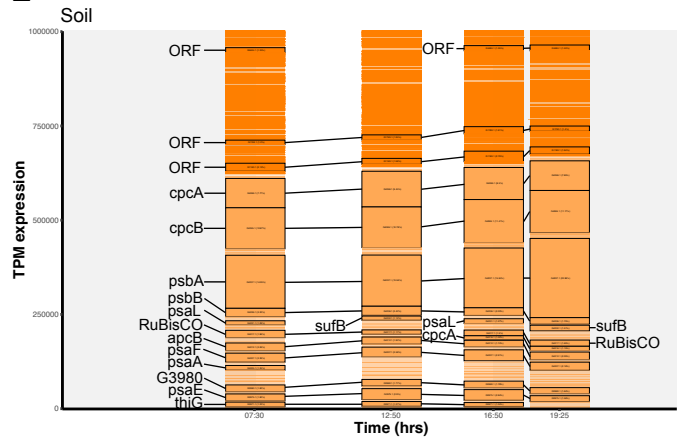

Supplement: Supplementary_Figure_7_ycae151 [file supplementary_figure_7_ycae151.pdf]

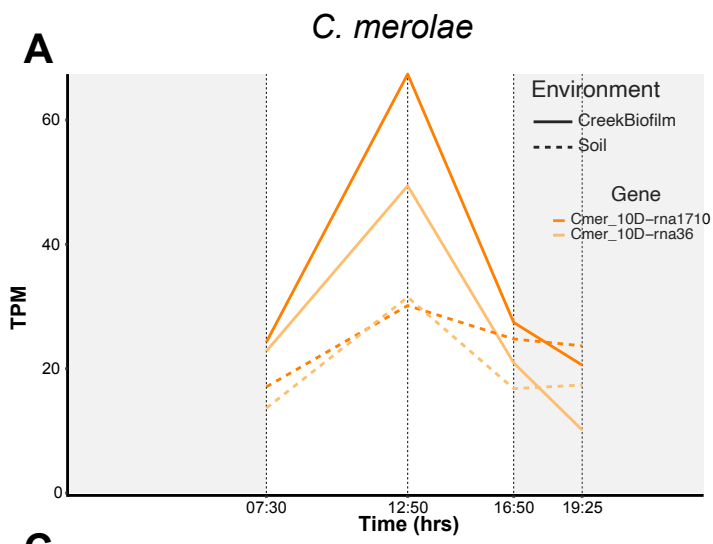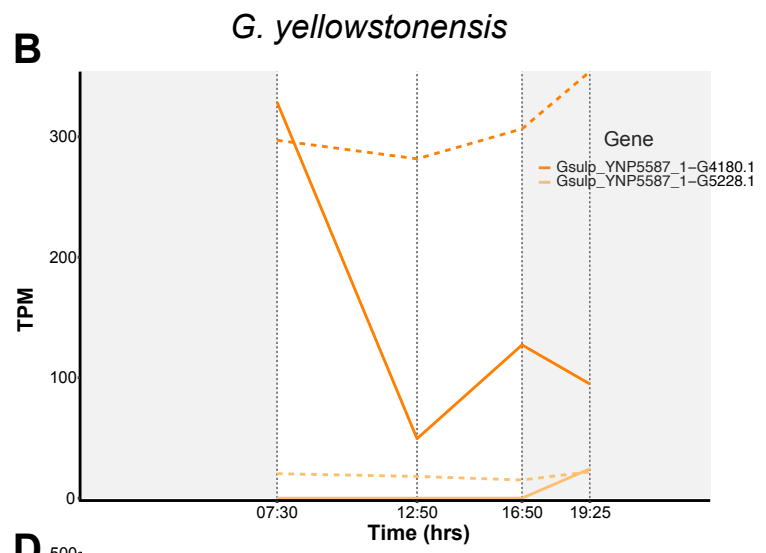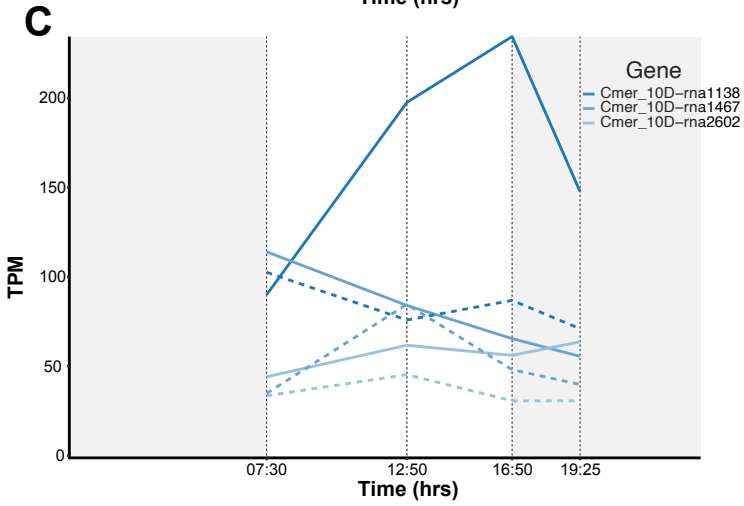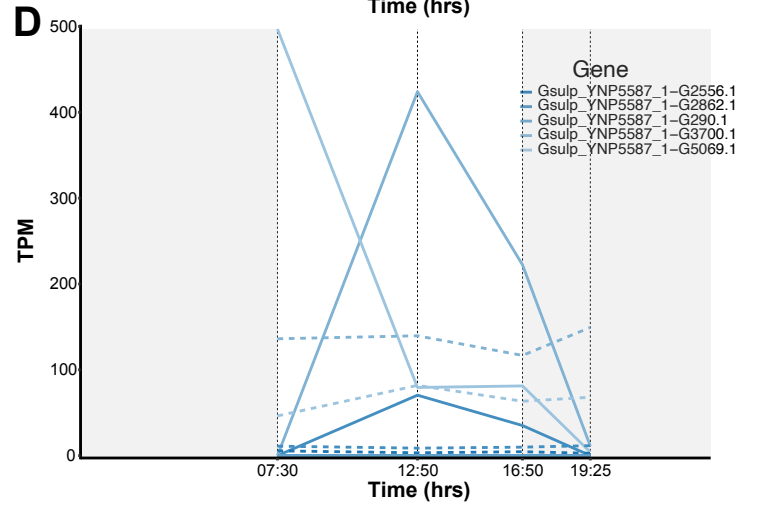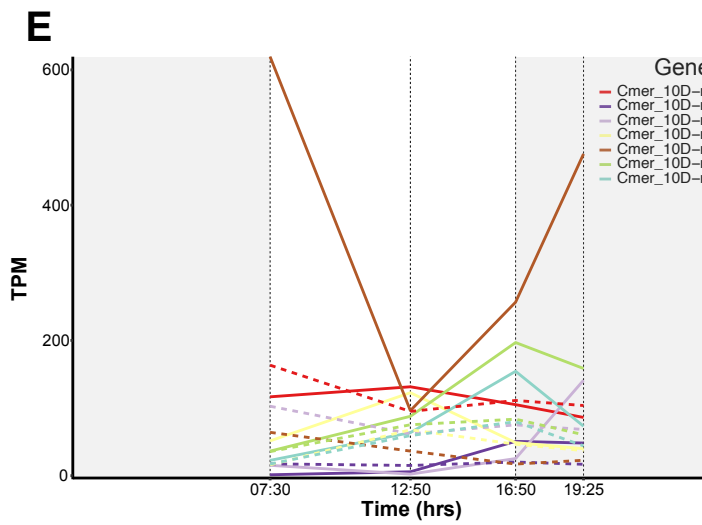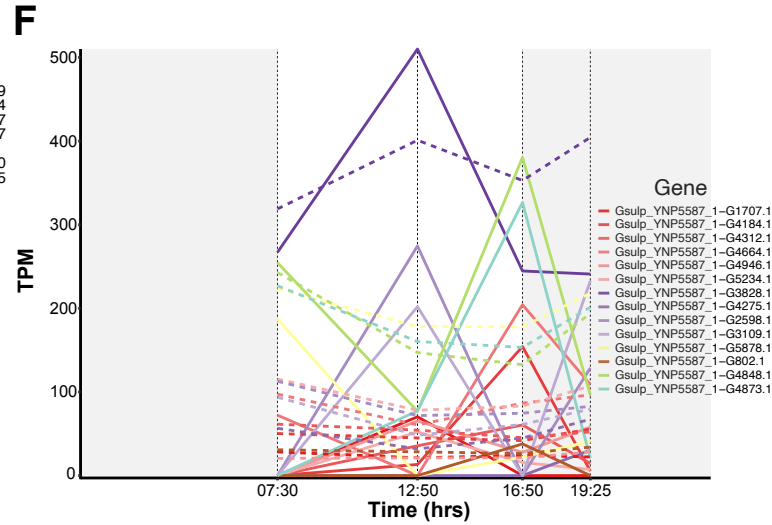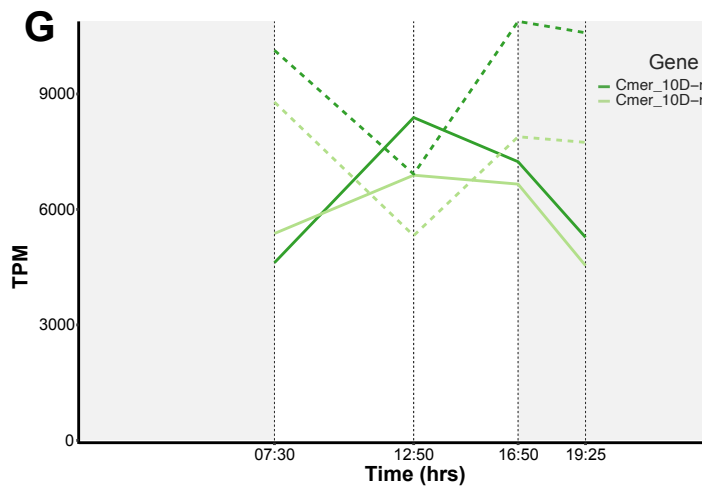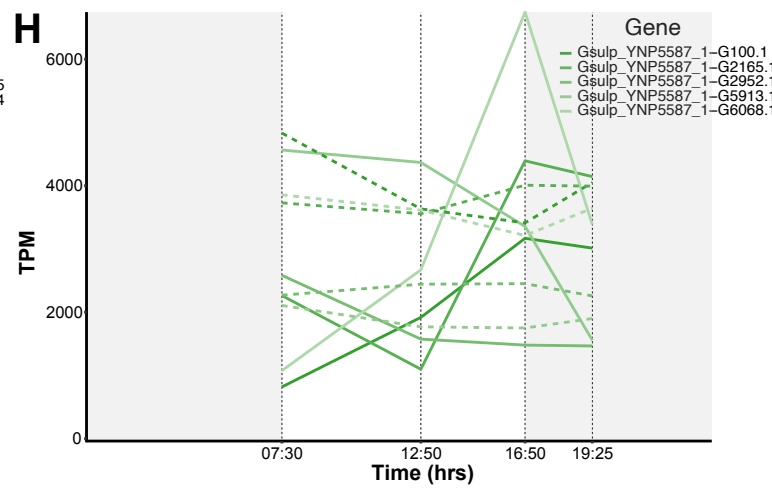

Supplement: Supplementary_Figure_9_ycae151 [file supplementary_figure_9_ycae151.pdf]

**A**

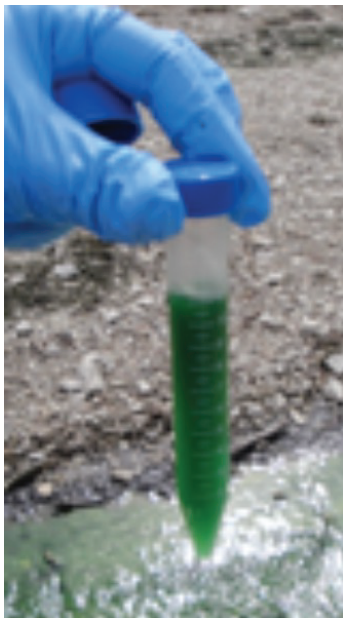

**B**

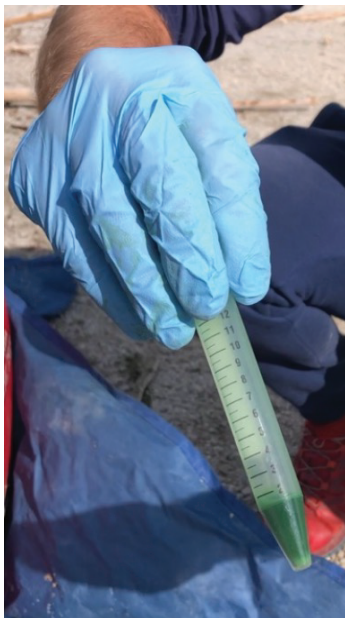

**C**

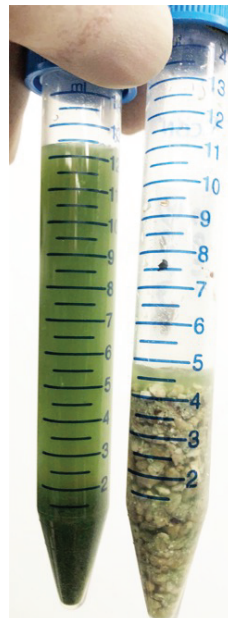

Supplement: Supplementary_Figure_10_ycae151 [file supplementary_figure_10_ycae151.pdf]

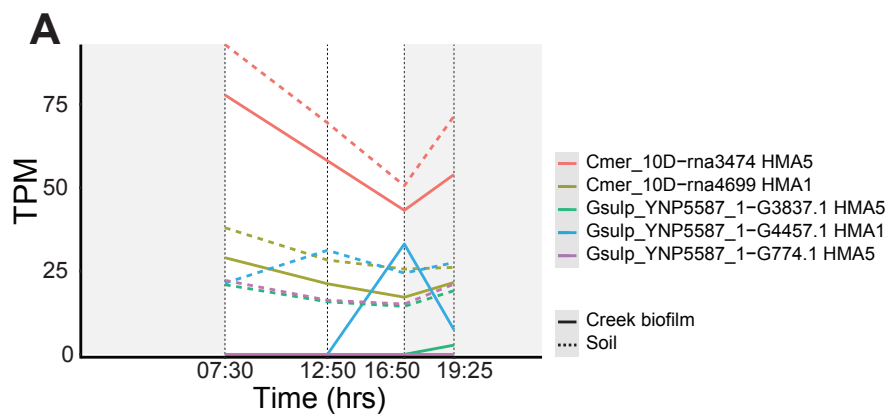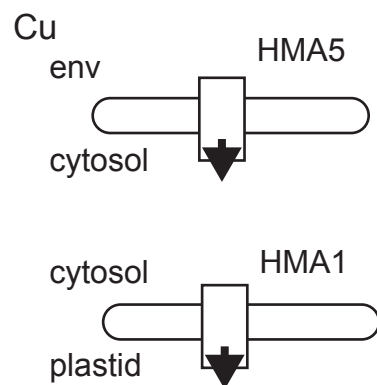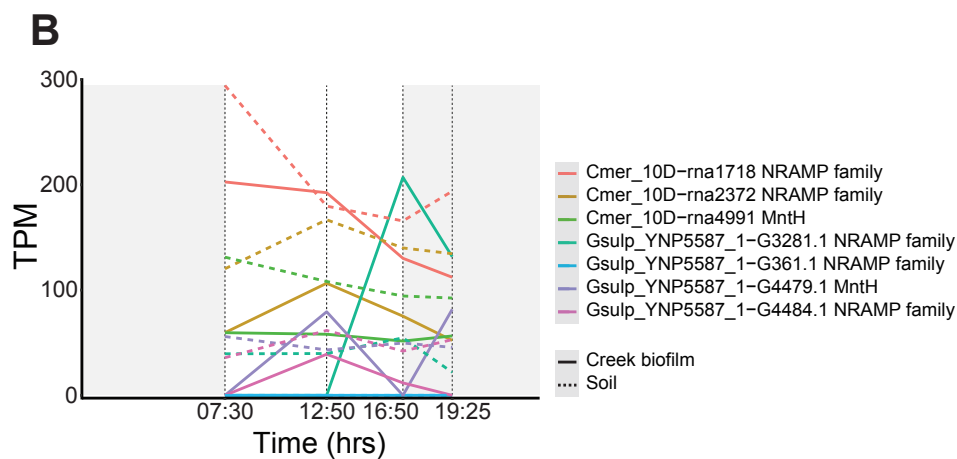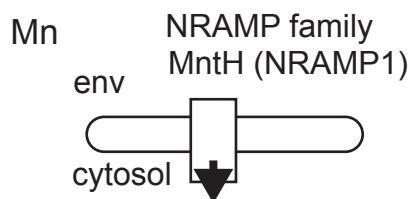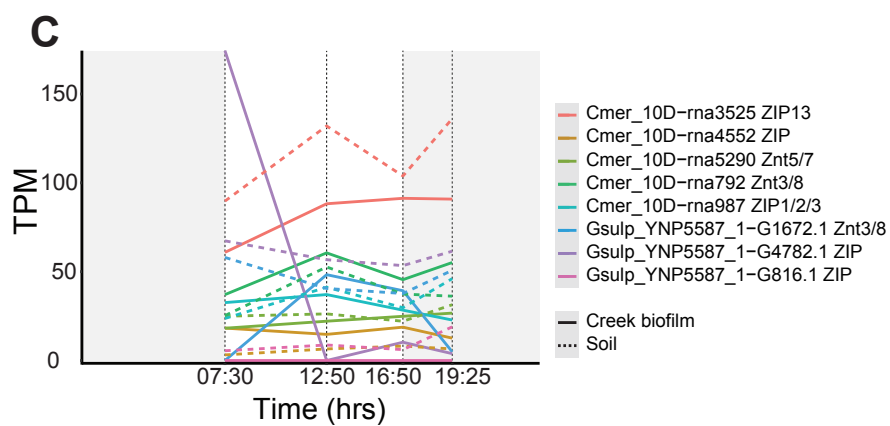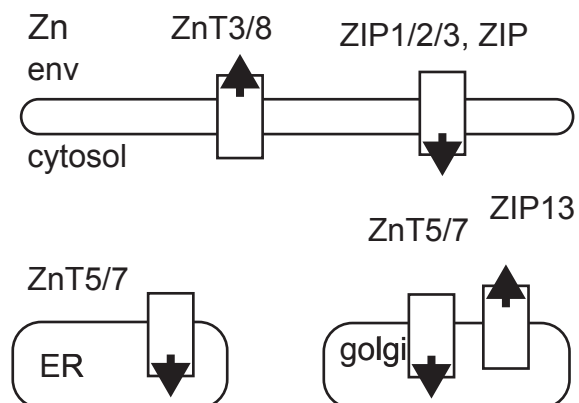

Supplement: Supplementary_Figure_11_ycae151 [file supplementary_figure_11_ycae151.pdf]

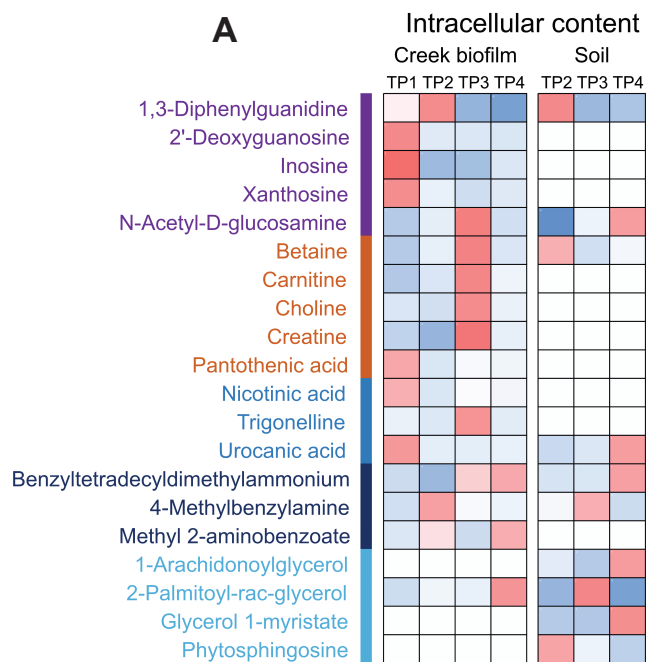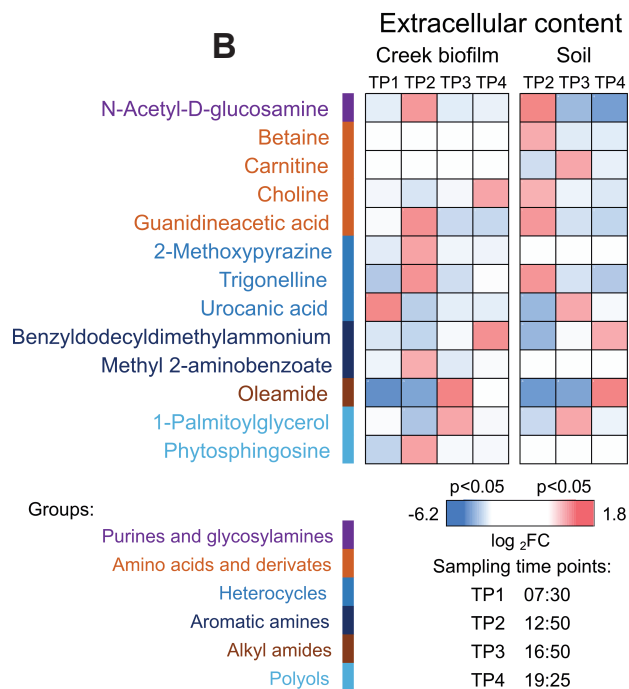

Supplement: Supplementary_Figure_12_ycae151 [file supplementary_figure_12_ycae151.pdf]
