# Supplementary material for: Temporal dynamics in a red alga dominated geothermal feature in Yellowstone National Park
Source: ISME Commun. 2024 Dec 3;4(1):ycae151. doi: 10.1093/ismeco/ycae151 (PMC11662350; doi:10.1093/ismeco/ycae151)
Supplement: Supplementary_Figure_6_ycae151 [file supplementary_figure_6_ycae151.pdf]

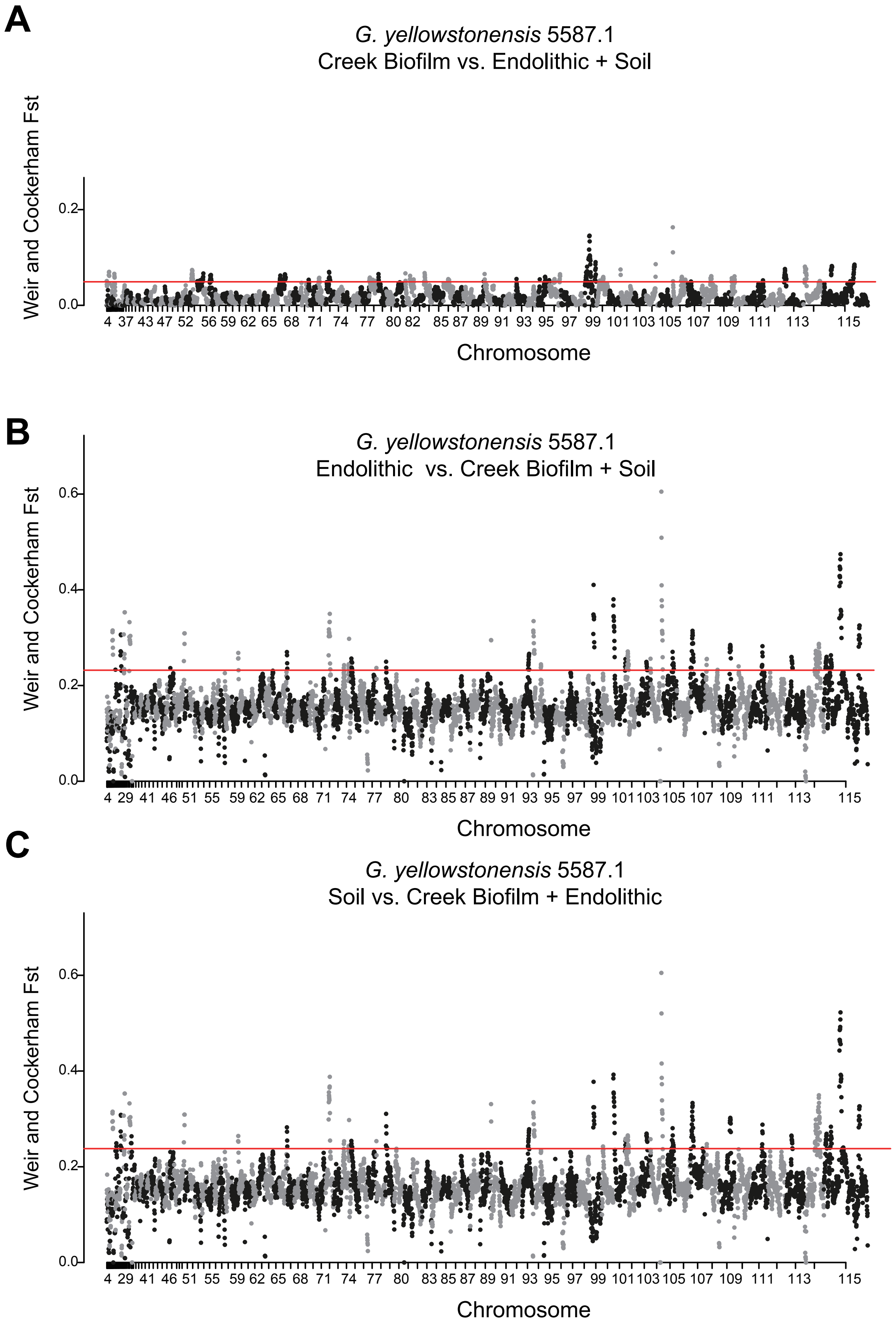
A
G. yellowstonensis 5587.1
Creek Biofilm vs. Endolithic + Soil
Weir and Cockerham Fst
0.0
0.2
4 37 43 47 52 56 59 62 65 68 71 74 77 80 82 85 87 89 91 93 95 97 99 101 103 105 107 109 111 113 115
Chromosome
B
G. yellowstonensis 5587.1
Endolithic vs. Creek Biofilm + Soil
Weir and Cockerham Fst
0.0
0.2
0.4
0.6
4 29 41 46 51 55 59 62 65 68 71 74 77 80 83 85 87 89 91 93 95 97 99 101 103 105 107 109 111 113 115
Chromosome
C
G. yellowstonensis 5587.1
Soil vs. Creek Biofilm + Endolithic
Weir and Cockerham Fst
0.0
0.2
0.4
0.6
4 29 41 46 51 55 59 62 65 68 71 74 77 80 83 85 87 89 91 93 95 97 99 101 103 105 107 109 111 113 115
Chromosome
