# Supplementary material for: Temporal dynamics in a red alga dominated geothermal feature in Yellowstone National Park
Source: ISME Commun. 2024 Dec 3;4(1):ycae151. doi: 10.1093/ismeco/ycae151 (PMC11662350; doi:10.1093/ismeco/ycae151)
Supplement: Supplementary_Figure_8_ycae151 [file supplementary_figure_8_ycae151.pdf]

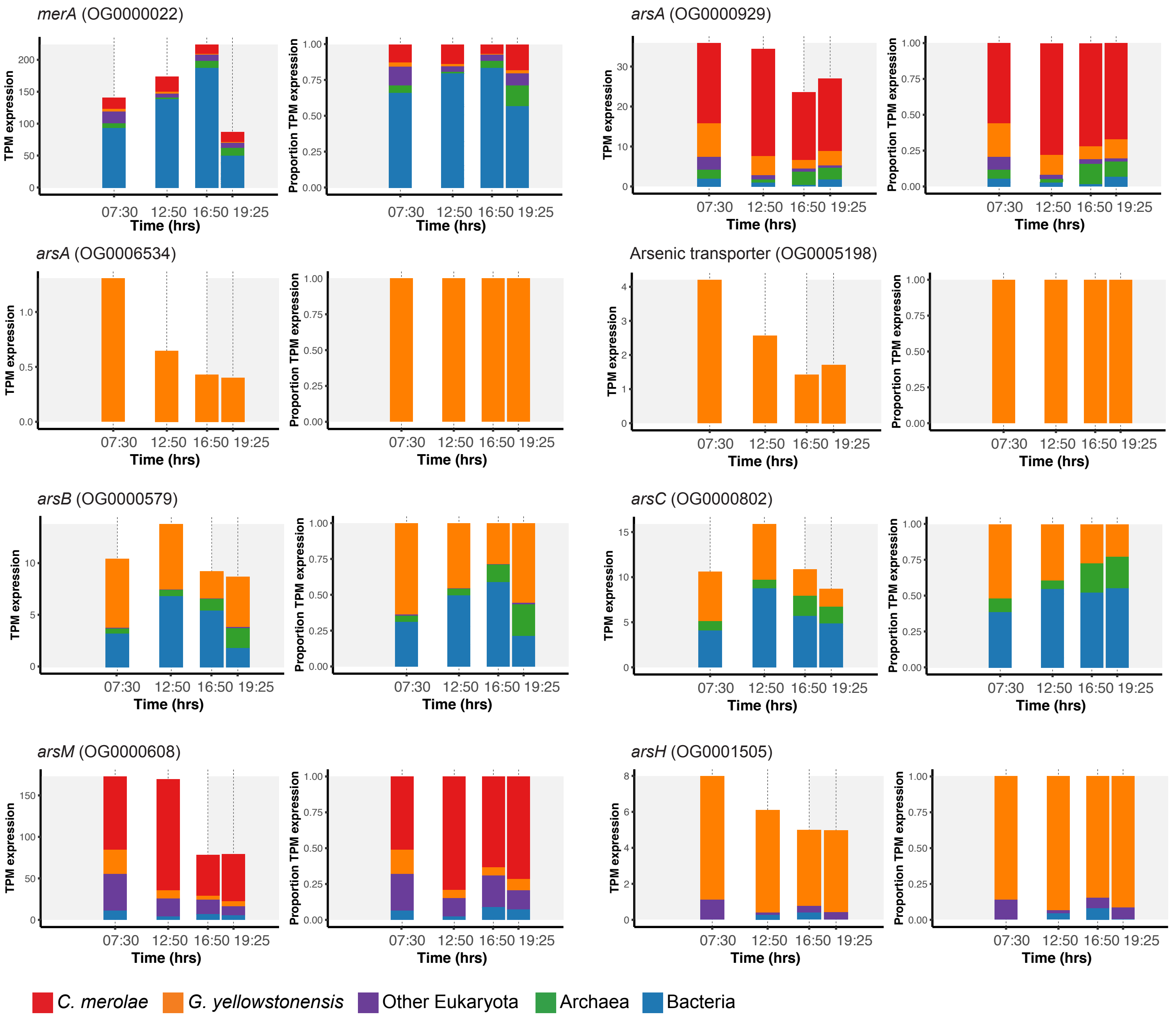
merA (OG0000022)
arsA (OG0000929)
arsA (OG0006534)
Arsenic transporter (OG0005198)
arsB (OG0000579)
arsC (OG0000802)
arsM (OG0000608)
arsH (OG0001505)
TPM expression
Proportion TPM expression
07:30 12:50 16:50 19:25
Time (hrs)
C. merolae
G. yellowstonensis
Other Eukaryota
Archaea
Bacteria
