# Supplementary material for: Temporal dynamics in a red alga dominated geothermal feature in Yellowstone National Park
Source: ISME Commun. 2024 Dec 3;4(1):ycae151. doi: 10.1093/ismeco/ycae151 (PMC11662350; doi:10.1093/ismeco/ycae151)
Supplement: Supplementary_Figure_14_ycae151 [file supplementary_figure_14_ycae151.pdf]

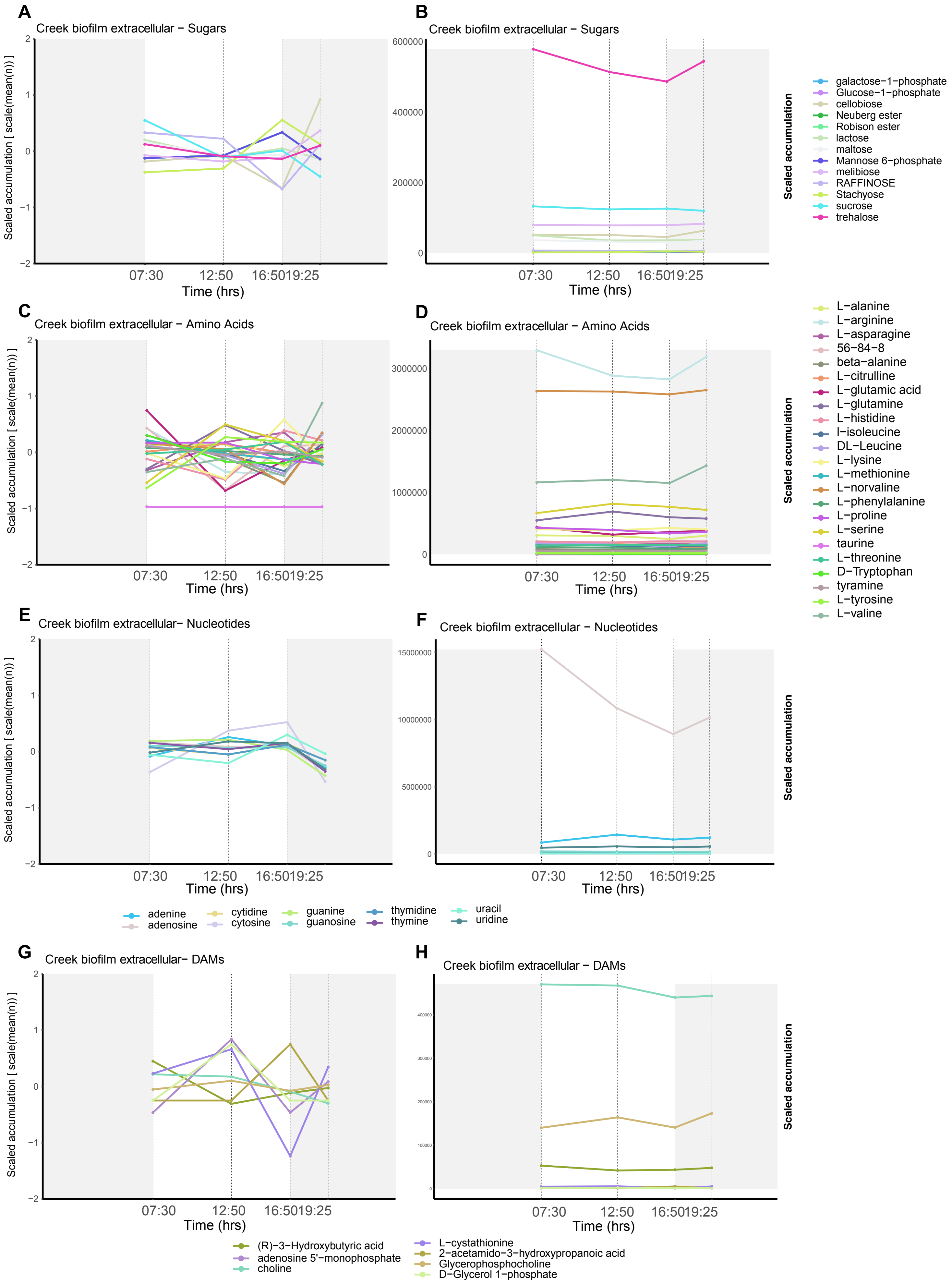
A
Creek biofilm extracellular − Sugars
Scaled accumulation [scale(mean(n))]
Time (hrs)
07:30
12:50
16:50
19:25
B
Creek biofilm extracellular − Sugars
Scaled accumulation
600000
400000
200000
0
galactose−1−phosphate
Glucose−1−phosphate
cellobiose
Neuberg ester
Robison ester
lactose
maltose
Mannose 6−phosphate
melibiose
RAFFINOSE
Stachyose
sucrose
trehalose
C
Creek biofilm extracellular − Amino Acids
D
Creek biofilm extracellular − Amino Acids
3000000
2000000
1000000
L−alanine
L−arginine
L−asparagine
56−84−8
beta−alanine
L−citrulline
L−glutamic acid
L−glutamine
L−histidine
l−isoleucine
DL−Leucine
L−lysine
L−methionine
L−norvaline
L−phenylalanine
L−proline
L−serine
taurine
L−threonine
D−Tryptophan
tyramine
L−tyrosine
L−valine
E
Creek biofilm extracellular− Nucleotides
F
Creek biofilm extracellular − Nucleotides
15000000
10000000
5000000
adenine
adenosine
cytidine
cytosine
guanine
guanosine
thymidine
thymine
uracil
uridine
G
Creek biofilm extracellular− DAMs
H
Creek biofilm extracellular − DAMs
400000
300000
200000
100000
(R)−3−Hydroxybutyric acid
adenosine 5'−monophosphate
choline
L−cystathionine
2−acetamido−3−hydroxypropanoic acid
Glycerophosphocholine
D−Glycerol 1−phosphate
